# Relating spidroin motif prevalence and periodicity to the mechanical properties of major ampullate spider silks

**DOI:** 10.1007/s00360-022-01464-3

**Published:** 2022-11-07

**Authors:** Joseph Arguelles, Richard H. Baker, Jose Perez-Rigueiro, Gustavo V. Guinea, M. Elices, Cheryl Y. Hayashi

**Affiliations:** 1 Division of Invertebrate Zoology and Institute for Comparative Genomics, American Museum of Natural History, New York, NY 10024 USA; 2 Center for Biomedical Engineering (CTB), Universidad Politécnica de Madrid, Pozuelo de Alarcón, 28223 Madrid, Spain; 3 Centro de Investigatión Biomédica en Red de Bioingeniería, Biomateriales y Nanomedicina (CIBER-BBN), Instituto de Salud Carlos III, Madrid, Spain; 4 Departamento de Ciencia de Materiales, Universidad Politécnica de Madrid, ETSI Caminos, Canales y Peurtos, 28040 Madrid, Spain; 5 Biomaterials and Regenerative Medicine Group, Instituto de Investigación Sanitaria del Hospital Clínico San Carlos (IdISSC), Calle Prof. Martín Lagos s/n, 28040 Madrid, Spain

**Keywords:** Alpha parameter, Dragline, Major ampullate spidroin, Periodicity, Silk, Spider, Spidroin, Supercontraction

## Abstract

Spider dragline fibers exhibit incredible mechanical properties, outperforming many synthetic polymers in toughness assays, and possess desirable properties for medical and other human applications. These qualities make dragline fibers popular subjects for biomimetics research. The enormous diversity of spiders presents both an opportunity for the development of new bioinspired materials and a challenge for the identification of fundamental design principles, as the mechanical properties of dragline fibers show both intraspecific and interspecific variations. In this regard, the stress–strain curves of draglines from different species have been shown to be effectively compared by the α* parameter, a value derived from maximum-supercontracted silk fibers. To identify potential molecular mechanisms impacting α* values, here we analyze spider fibroin (spidroin) sequences of the Western black widow (*Latrodectus hesperus*) and the black and yellow garden spider (*Argiope aurantia*). This study serves as a primer for investigating the molecular properties of spidroins that underlie species-specific α* values. Initial findings are that while overall motif composition was similar between species, certain motifs and higher level periodicities of glycine-rich region lengths showed variation, notably greater distances between poly-A motifs in *A. aurantia* sequences. In addition to increased period lengths, *A. aurantia* spidroins tended to have an increased prevalence of charged and hydrophobic residues. These increases may impact the number and strength of hydrogen bond networks within fibers, which have been implicated in conformational changes and formation of nanocrystals, contributing to the greater extensibility of *A. aurantia* draglines compared to those of *L. hesperus*.

**Supplementary Information:**

The online version contains supplementary material available at 10.1007/s00360-022-01464-3.

## Introduction

In materials science, researchers often turn to biomimetics, drawing inspiration from nature to improve upon their own designs. Spider silk, a popular target for biomimicry, is a natural polymer with an impressive range of mechanical properties that have evolved over several hundred million years. Among the different types of silks spun by spiders, the most widely studied is major ampullate (MA) silk, which is used as a safety dragline when rappelling and as the main structural component of orb-web frames. MA silks are both strong and extensible, resulting in fibers with toughness that outclasses man-made materials such as Kevlar and steel (Gosline et al. 1999). MA silk fibers are also naturally resistant to microbial growth (Wright and Goodacre 2012), elicit no immune response when implanted surgically (Herold et al. 2020), and are effective scaffolds for the regeneration of severed nerves (Kornfeld, 2021). This combination of qualities makes MA silk a highly valuable material for both engineering and medicine, leading to increasing interest in their mass-production. Spiders, however, are territorial and cannibalistic, making large-scale farming impractical. Research has, as a result, turned toward reproducing spider silk fibers artificially. While several transgenic and synthetic silks have been developed (Greco et. al 2020; Andersson et al. 2017; Gonska et. al 2020; Venkatesan et. al 2019), it remains difficult to fully replicate the desirable mechanical properties of their natural counterparts, mainly due to significant gaps in our understanding of their fundamental design principles.

To better understand how spider silks achieve their extraordinary mechanical properties, genomic methods can provide insight into the molecular basis for spider silks. Spider silks have been found to be largely composed of proteins belonging to the spidroin (spider fibroin) family. Spidroin sequences tend to be very long (e.g., 3000 amino acids; Hinman et al. 1992; Ayoub and Hayashi 2007) and consist of strongly conserved amino (*N*)- and carboxyl (C)-terminal regions flanking a highly repetitive central region (Garb et al. 2010; Xu and Lewis 1990). Unlike the N- and C-terminal regions, the sequences of the highly repetitive central regions vary greatly across spidroin family members (Starrett et. al 2012; Babb et. al 2017). Comparative analyses of the repetitive regions of major ampullate spidroin (MaSp) sequences have identified amino acid motifs that are believed to confer structural and mechanical properties to MA silk fibers. Most generally, poly-alanine (poly-A) blocks within the repetitive regions have been demonstrated to assemble during silk extrusion, forming crystalline beta-sheets responsible for the fluid–solid transition required to spin the material. These poly-A blocks are also thought to bolster the overall strength of silks (Xu and Lewis, 1990). The other major component of the central repetitive region are large stretches enriched with glycine-rich motifs, such as GGX (where *X* = A, L, Q, Y, R), GXG (where *X* = L, R, Q, Y, P), and poly-GA, which form amorphous coiled structures, conferring extensibility to silks fibers (Yarger et al. 2018; Hayashi et al. 1999). Combined, the occurrence of motifs, and their variability between species, can explain how spider silks have been readily and widely modified by evolutionary processes to serve a range of mechanical needs. However, the large size, highly repetitive nature, and sequence variability of one MaSp gene to the next have made attempts to quantify and compare their molecular complexity difficult.

Recently, advances have been made in classifying the mechanical properties of dragline silks, by taking advantage of its unique ability to supercontract. Supercontraction of dragline silks occurs when the fibers are exposed to water, triggering rapid shrinkage up to ~ 50% of their length, an attribute that allows the fibers to return to a base-level after load-bearing (Elices et al. 2004; Blamires et al. 2012). As such, the supercontracted condition of dragline silks can be interpreted as a silk “ground state” that can be leveraged for making comparisons between MA silk fibers. Thus, by forcing MA silk fibers to achieve the maximum-supercontracted (MS) state before tensile testing, the intraspecific variability of silk mechanical properties (Madsen et al. 1999), such as those arising from sheer force during the spinning process, can be reduced to a negligible level (Madurga et al. 2016). Thus, it is possible to obtain a reliable quantitative classification of the stress–strain curves of MA fibers. This method utilizes a reference stress–strain curve (averaged from stress–strain curves of MS-MA fibers of the black and yellow garden spider, *Argiope aurantia*), and comparing this reference curve with the stress–strain curve obtained by tensile testing MS-MA silk fibers of other spider species of interest. Such comparisons have shown that by displacing the true stress-true strain curves along the true strain axis (X axis), the curves concur at high values of strain. The amount by which the curve of the fiber of interest is displaced along the strain axis defines the α* parameter and offers a simple procedure for the quantitative classification of the mechanical properties of MA silk fibers (Garrote et al. 2020).

With the ability to compare α* values between species, we are now poised for more targeted analyses of spidroins to better understand how differences in spidroin sequences relate to the structural and mechanical differences between silks. Molecular interactions, such as hydrogen bond formation and disulfide bridges, impact the mechanical potential of dragline fibers, from protein–protein association during fiber formation (Römer and Scheibel 2008) to the shifting and formation of crystalline structures during load-bearing (Madurga et al. 2016) and supercontraction (Cohen et al. 2021). These types of molecular interactions hinge largely upon the spatial relationships of participating amino acids. The repetitive nature of spidroins presents a particularly interesting case, in which the participating residues and the motifs they reside in occur in higher level patterns that are often well conserved throughout the length of the protein sequence. The prevalence of repeat structure among diverse spidroin types suggests that this configuration has functional significance to the general performance of silk. To this end, we focus on quantifying the prevalence and higher level periodicity of motifs in the repetitive regions noted for their potential role in influencing dragline fiber mechanical properties, namely GXG, GGX, poly-A, poly-GA, QQ, and SQ. It is important to note, however, that there are other factors that may also influence mechanical potential, such as post-translational modifications, overall spidroin composition of spun fibers, or fiber arrangement and microstructure. As the α* parameter is a recent advancement, few species exist where both α* value and full-length spidroin sequence data, necessary for analysis of the repetitive regions, are available. Here, we analyze the MaSp sequences of *A. aurantia* and *Latrodectus hesperus* (Western black widow), two species with different dragline α* values (0.00 and 0.53, respectively) and full-length spidroin sequence data, providing an in-depth look at the molecular variance in their sequences. The higher α* value of the *L. hesperus* dragline indicates that the silk fiber undergoes less extension before transitioning to the stiffening phase than the *A. aurantia* dragline. Through comparative analyses, we have identified several motifs of interest as possible candidates for explaining the differences in α* values of the MA silk fibers in both species, including some less-discussed motifs (e.g., QQ/SQ). We also identify other factors potentially contributing to these differences, particularly the overall periodicity of glycine-rich region length and the presence and periodicities of both charged and hydrophobic residues within spidroin sequences.

## Methods

### Spidroin sequences

Major ampullate spidroins (MaSps) of *Latrodectus hesperus* spiders were downloaded from NCBI GenBank and for *Argiope aurantia* they were obtained from Baker et al. (unpublished). For each class of MaSp (e.g., MaSp1, MaSp2, MaSp3) in *A. aurantia* full-length sequences were used to investigate a wide range of MaSp sequence variation. The *A. aurantia* GenBank accession numbers are: MaSp1a (ON722379), MaSp1b (ON722375), MaSp2.1a (ON722368), MaSp2.1b (ON722369), MaSp2.2a (ON722372), MaSp2.2b (ON722370), MaSp2.2c (ON722367), MaSp2.2d (ON722366) MaSp2.2e (ON722371), MaSp3a (ON722378), MaSp3b (ON722377). For *L. hesperus*, full-length sequences representing MaSp1 (ABR68856) and MaSp2 (ABR68855.1) were analyzed. *L. hesperus* MaSp3 was not included in this study because only partial-length sequences were available (AWK58730, AWK58638.1).

### Motif counts

**Fig. 1:**
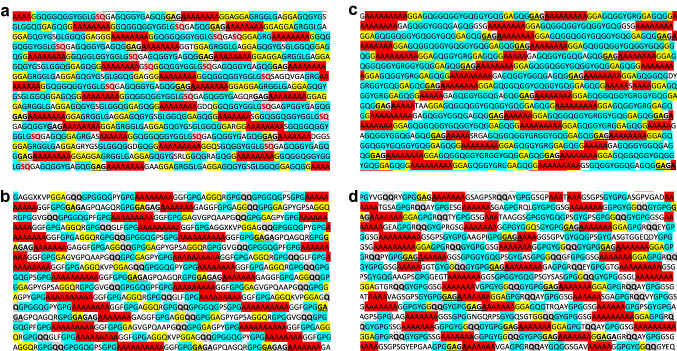
Visualization of motifs in major ampullate spidroins (MaSps). **a** A*rgiope aurantia* MaSp1a, **b** *Argiope aurantia* MaSp2.2a, **c** *Latrodectus hesperus* MaSp1, and **d** *Latrodectus hesperus* MaSp2 sequences with motifs colored (red, poly-A; blue, GXG; yellow, GGX; grey with red text, SQ; grey with black text, QQ; bold and underlined, poly-GA). Spidroins are shown in part. Complete spidroins with motif colorings are in Supplementary Fig. 6

Motif occurrences (GXG, GGX, poly-A, poly-GA, QQ, SQ) were identified in spidroin sequences and for each motif, the total number of participating residues was counted. These motif types were chosen for analyses as they have been noted in literature to be likely candidates for impacting the overall mechanical performance of spider dragline fibers (Yarger et al. 2018; Hayashi et al. 1999). In the case of poly-A and poly-GA motifs, motifs were only counted if they occurred in stretches of at least three residues and four residues in length, respectively. This process was repeated for each motif, per spidroin, and total residue counts were recorded, as illustrated in Fig. 1. These raw counts were then used to calculate the total percentage of the full-length sequence (including terminal regions) occupied by each motif. The motifs were then ranked based upon these raw percentages, from highest contribution to lowest. This process was repeated for each spidroin sequence.

### Quantifying overlap and motif percentage corrections

In some cases, such as GXG/GGX or poly-GA/poly-A, adjacent motifs overlapped leading to double-counting of participating residues (e.g., the underlined residues in GYGYGGAAAAA). To account for this, residues participating in multiple motifs simultaneously were identified (Supplemental Table 1). Corrections were made by subtracting the total overlap from the less dominant motif, as determined by the ranking of raw percentages. In the case of overlapping with a poly-A motif all residues were attributed to the poly-A count, regardless of ranking, as the poly-A motif’s formation of crystalline *β*-sheets prompts the transition from protein solution to solid fiber characteristic of the spinning process. These newly corrected values were then used to determine the overall percent contribution of each motif, relative to the entire sequence.

### Uninterrupted motif lengths

Motifs were identified as described above. Often, the same motif occurs consecutively without interruption within a repeat unit (e.g., GYGYGQGYGQG for the GXG motif). We recorded the length of all instances of these concurrent motif runs for all motif types and spidroin sequences (Table 1, Supplemental Fig. 1).

**Table 1:** Mean motif lengths of major ampullate spidroins

Spidroin	Poly-A	GGX uninterrupted	GXG uninterrupted
*L. hesperus* MaSp1	7.6	3.6	7.3
*A. aurantia* MaSp1a	7.7	4.8	5.9
*A. aurantia* MaSp1b	6.3	4.1	3.8
*L. hesperus* MaSp2	7.0	3.0	4.1
*A. aurantia* MaSp2.1a	6.2	3.4	4.4
*A. aurantia* MaSp2.1b	6.9	3.3	4.6
*A. aurantia* MaSp2.2a	8.6	3.0	3.5
*A. aurantia* MaSp2.2b	7.8	3.0	4.1
*A. aurantia* MaSp2.2c	7.8	3.0	4.1
*A. aurantia* MaSp2.2d	7.6	3.0	4.2
*A. aurantia* MaSp2.2e	6.7	3.0	4.0
*A. aurantia* MaSp3a	5.1	3.0	4.8
*A. aurantia* MaSp3b	4.1	3.1	3.8

Mean lengths (in residues) of poly-A motifs and mean uninterrupted occurrences of GGX and GXG are shown for MaSp sequences from *Latrodectus hesperus* and *Argiope aurantia*

### Periodicity of glycine-rich regions

The average lengths of glycine-rich regions between poly-A motifs varied from spidroin to spidroin, suggesting that overall periodicity of motifs may be of importance. Spidroin sequences were divided into repeat segments between each poly-A motif. The glycine-rich segments, directly following each poly-A, were counted and their lengths were recorded.

## Results and discussion

### Motif coverage and composition of major ampullate spidroins

**Fig. 2:**
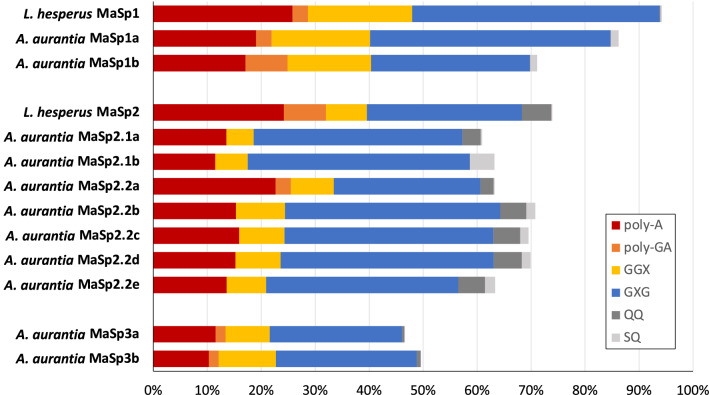
Motif percentage of major ampullate spidroins. Percentage of each of seven motifs in major ampullate spidroins of *Argiope aurantia* and *Latrodectus hesperus.* Complete motif breakdown (including all GGX and GXG variants) is available in Supplemental Fig. 2

To better understand the molecular underpinnings of α*, a quantitative parameter for the classification of major ampullate silk mechanical properties, we analyzed the MaSp sequences of *A. aurantia* and *L. hesperus* (α* = 0.00 and 0.53, respectively; Madurga et al. 2016). We focused on the repetitive regions, which consist of iterated combinations of various amino acid motifs (e.g., poly-A, GXG, GGX). Considering MaSp1, both *L. hesperus* MaSp1 and *A. aurantia* MaSp1a were found to have similar values for overall motif coverage, 94.2% and 86.2% coverage, respectively (Fig. 2). *A. aurantia* MaSp1b, however, had a lower overall motif coverage, 71.1%. For MaSp2, again, the motifs accounted for well over half of both *L. hesperus* and *A. aurantia* sequences, ranging from 60.9% (*A. aurantia* MaSp2.1a) to 73.9% (*L. hesperus* MaSp2). By contrast, MaSp3a and MaSp3b of *A. aurantia* were not as dominated by motifs as MaSp1 and MaSp2 sequences, with overall motif coverages of just 46.6% and 49.6%, respectively. As the stereotypical motifs used to describe MaSp sequences were developed prior to the discovery of MaSp3 (Collin et al. 2018), this lower overall coverage is unsurprising and does not necessarily imply a less functional role of this MaSp type. It is expected that our recognition of motifs, as they relate to spidroins, will expand with increased understanding of spidroin diversity. This, however, will require additional analyses of these newly identified MaSps to better understand which repetitive sequence elements are shared widely among members of this MaSp type and should be considered stereotypical motifs.

Given that the overall motif coverage was similar between MaSp1 and MaSp2 sequences of *L. hesperus* and *A. aurantia* (Fig. 2), motif coverage percentage appears not to explain the difference in their α*. We next examined motif composition of the repetitive regions to determine if the prevalence of specific motifs could be responsible for the variation in dragline silk properties. *A. aurantia* MaSp1b differed from other MaSp1 sequences (Fig. 2, Supplemental Fig. 2), showing both higher poly-GA coverage (7.8%, ~ twice that of either *L. hesperus* MaSp1 or *A. aurantia* MaSp1a) and lower GXG motif coverage (29.4%), which is a ~ 33% reduction compared to *L. hesperus* MaSp1 (45.9%) and *A. aurantia* MaSp1a (44.5%). GGX motif coverage was similar across species, with coverage ranging from 15.5% (*A. aurantia* MaSp1b) to 19.3% (*L. hesperus* MaSp1). In terms of specific motif types, *A. aurantia* MaSp1s had relatively high representation of the GLG motif (9.9%, MaSp1a; 15.2% MaSp1b), but this motif is entirely absent in *L. hesperus* MaSp1. Similarly, differences were observed in the abundance of the SQ motif. *A. aurantia* MaSp1 sequences had a mean sequence coverage 1.4% (1.5% MaSp1a; 1.3% MaSp1b) of the SQ motif which although modest, was nearly absent in *L. hesperus* MaSp1 (0.3%) (Figs. 1, 2). As both S and *Q* residues are polar, it may be that their presence in the repetitive regions of *A. aurantia* spidroin sequences allows for biochemical interactions that provide *A. aurantia* fibers with increased toughness through hydrogen bonding (Blackledge et al. 2012).

MaSp2 sequences showed more variation in overall motif composition, both between species and within *A. aurantia* MaSp2 spidroins (Fig. 2). Most dramatic was the near-total absence of poly-GA motifs from *A. aurantia* MaSp2 sequences (mean: 0.4%, range 0.0–2.9%), with only MaSp2.2a having an appreciable amount (2.9%), compared to the 7.8% in *L. hesperus*. GGX motifs, which consisted mostly of the GGA motif type after overlap correction, accounted for 5.0% (MaSp2.1a) to 9.1% (MaSp2.2b) in *A. aurantia*, the average of which was similar to the 7.6% in *L. hesperus* MaSp2. GXG motif coverage ranged from 27.2% (MaSp2.2a) to 41.1% (MaSp2.1b) of overall sequence in *A. aurantia* (mean: 37.2%), and coverage in *L. hesperus* MaSp2 (28.7%) was within that range.

Considering specific GXG motif types, the GYG motif was nearly absent from *A. aurantia* MaSp2.2a (0.2%), but well represented in the other *A. aurantia* MaSp2 sequences (mean: 5.9%, range 3.0–11.1%) and *L. hesperus* MaSp2 (7.8%). GPG, a special class of GXG motif seen most abundantly in MaSp2 sequences, has been shown to impact the extensibility of MA fibers (Savage and Gosline 2008a, b; Liu et al. 2008; Rauscher et al. 2006; Creager et al. 2010; Perez-Rigueiro et al. 2021). In *L. hesperus* MaSp2, GPG motifs were less predominant (19.0%) than in *A. aurantia* MaSp2s (mean: 26.5%, range 20.9–28.8%).

*L. hesperus* MaSp2 sequences also showed increased poly-A contribution (24.2%), an ~ 60% increase compared to *A. aurantia* MaSp2 sequences (mean: 15.4%, range with MaSp2.2a excluded: 11.4–15.9%), with the singular exception of MaSp2.2a (22.7%). *L. hesperus* MaSp2s also had near-total absence of the SQ motif, utilizing a similar QQ motif instead (a trait shared with *A. aurantia* MaSp2.1a and MaSp2.2a; Fig. 2). This may indicate that the SQ motifs evolved in a recent ancestor of *Argiope* spiders, and that the inclusion and variation of the SQ/QQ motifs within MaSps may play a role in the interspecific variation of *Argiope* dragline mechanical properties (Malay et al. 2017; Jung et al. 2019). Notably, *A. aurantia* MaSp2.1b has just a single QQ motif but the highest SQ motif coverage (4.5%) of all sequences further suggesting QQ and SQ motifs may be serving similar functions. SQ/QQ motifs appear to be spider-specific, absent in fibroin sequences found in other silk-spinning arthropods, such as Trichoptera and Lepidoptera. Strikingly, they are also largely absent in *L. hesperus* MaSp1, but present in both *A. aurantia* MaSp1 sequences. These polar residues, with fairly regular occurrence throughout the sequence, could be forming hydrogen bonds when exposed to water, participating in the reorganization of the protein and, subsequently its supercontraction.

### Potential influence of motif length and periodicity on mechanical properties

In addition to the differences in motif composition between *L. hesperus* and *A. aurantia* MaSp sequences, we looked more closely at the uninterrupted lengths of the various motifs. Motifs such as GGX often occur successively (e.g., GGAGGQGGQGGY…) before being interrupted by a different motif. Longer stretches of uninterrupted motifs may have an additive or multiplicative effect on their derived mechanical properties. We found that uninterrupted poly-A and GGX length in MaSp1 sequences were similar across species, but there were some slight differences in the range of uninterrupted GXG motif lengths (3.8 *A. aurantia* MaSp1b–7.3 *L. hesperus* MaSp1) than did GGX (3.5 *L. hesperus* MaSp1–4.8 *A. aurantia* MaSp1a; Table 1). Motif lengths in MaSp2 sequences were also overall similar, both between species and within *A. aurantia*, except for a slightly longer poly-A runs in *A. aurantia* MaSp2.2a (mean length of 8.6 versus 6.0–7.8 amino acids in other sequences). MaSp3s from *A. aurantia* were distinct from MaSp1 and MaSp2 sequences in possessing the shortest average poly-A length (mean: 4.6 MaSp3s; mean: 7.3 MaSp2s; mean: 7.0 MaSp1s).

**Fig. 3:**
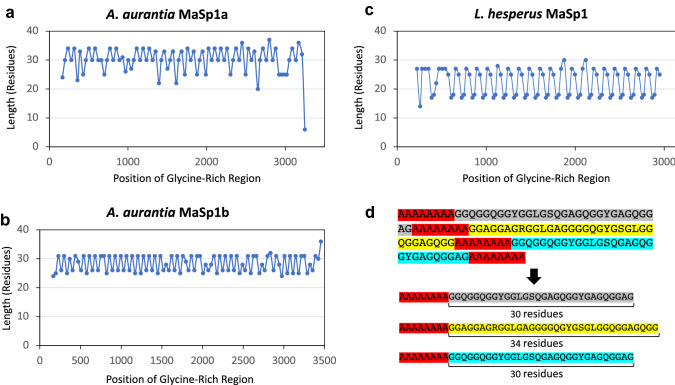
Periodicity of MaSp1 glycine-rich regions. Length of glycine-rich regions within **a** *Argiope aurantia* MaSp1a*,* **b** *Argiope aurantia* MaSp1b, and **c** *Latrodectus hesperus* MaSp1 sequences are plotted in order of occurrence. Points represent the length (in residues) of an individual region. **d** Schematic of dividing repetitive sequence into individual regions demarcated by poly-A regions and the counting of residues within each glycine-rich region

Thus, there were only subtle differences in uninterrupted motif lengths between the MaSp sequences from the two species, suggesting that motif array size alone is unlikely to be sufficient for explaining the variation in MaSp properties. We next considered higher level periodicity. The repetitive regions of *L. hesperus* MaSp sequences consist of multiple, conserved ensemble repeat units, in which smaller variable repeat units are nested within a larger repeat unit (Ayoub and Hayashi 2008). Investigating the glycine-rich regions, we noticed that not only did their overall lengths vary between species, but the occurrence of these regions (when classified by length) often displayed a higher level periodicity. In MaSp1 sequences, this is strongly the case for *L. hesperus*, while *A. aurantia* MaSp1 sequences displayed a less clear pattern (Fig. 3). On average, the lengths of glycine-rich regions were longer in *A. aurantia* MaSp1 sequences than in *L. hesperus* (mean lengths of 28.5 residues and 21.5 residues, respectively). Reporting average lengths, however, obscures that the lengths of glycine-rich regions appear in a highly conserved, recurring pattern in *L. hesperus* MaSp1 ([17-18-27-25]–[17-18-27-25]–[17-18-27-25]-…; averaging 21.5 residues; Fig. 3c). In *A. aurantia* MaSp1 sequences, there was some evidence of a conserved pattern of glycine-rich region lengths (such as stretches of region lengths [31-25/26]-[31-25/26]-[31-25/26]-… in *A. aurantia* MaSp1a and [31–25/26]-[31–25/26]-[31–25/26]-… in MaSp1b), but these patterns were frequently interrupted by periods of shorter length (Fig. 3a, b).

**Fig. 4:**
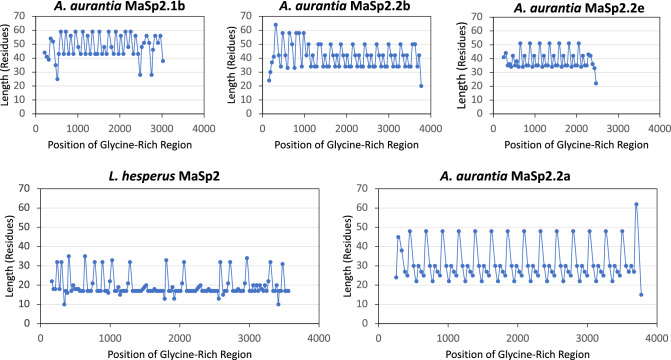
Periodicity of MaSp2 glycine-rich regions. Glycine-rich regions of *Argiope aurantia* and *Latrodectus hesperus* full-length MaSp2 sequences in order of occurrence. Points represent the length (in residues) of an individual region. Graphs grouped by similarity in overall motif coverage (Top: *A. aurantia* MaSp2.1b, MaSp2.2b and MaSp2.2e. Bottom: *L. hesperus* MaSp2 and *A. aurantia* MaSp2.2a). All MaSp2 graphs are available in Supplemental Fig. 3

*A. aurantia* MaSp2s showed a more obvious, large-scale pattern of glycine-rich region lengths (Fig. 4, all MaSp2 sequences in Supplemental Fig. 3). The comparison of *A. aurantia* and *L. hesperus* MaSp2 sequences, again, showed different overall lengths of glycine-rich regions, with those of *A. aurantia* largely 25–50 residues long, while *L. hesperus* regions were much shorter, mostly 17 residues long. While *A. aurantia* MaSp3s had no direct comparison in *L. hesperus*, their glycine-rich region lengths were more similar to *A. aurantia* MaSp1s (on average ranging from 21 to 31 aa) than MaSp2s. MaSp3b also showed glycine-rich region periodicity similar to *A. aurantia* MaSp2 sequences. This large-scale periodicity of MaSp glycine-rich regions highlights a clear contrast between species, with *L. hesperus* maintaining a stricter periodicity in MaSp1 sequences than *A. aurantia*, and vice-versa for MaSp2 sequences. This difference may indicate a significant role of overall periodicity in conferring toughness to silk fibers but will require more investigation, particularly extensions to additional species as α* values and full-length spidroin sequences become available. For instance, increased variability in the glycine-rich region period lengths of *A. aurantia* spidroins may contribute to the availability of “hidden length” within fibers, a process where new crystalline structures are formed in amorphous regions as hydrogen bonds break under stress, resulting in increased extensibility and toughness (Tarakanova and Buehler 2012a, b; Madurga et al. 2016).

### Unique characteristics of *A. aurantia*-specific silks

**Fig. 5:**
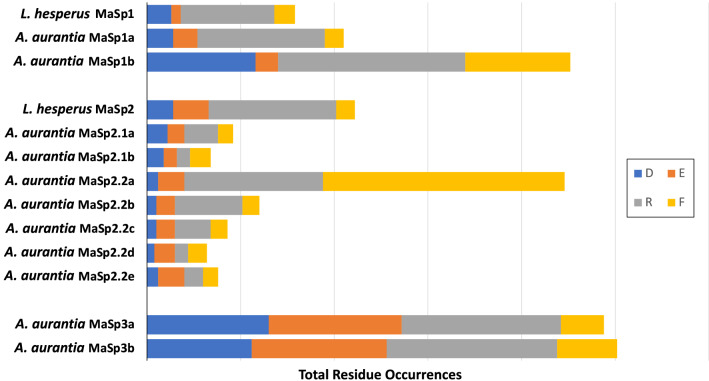
Prevalence of biochemically significant residues in *Argiope aurantia* and *Latrodectus hesperus* MaSps. Relative proportions of hydrophobic (Phe, orange), and charged (Arg, blue; Asp, grey) residues in full-length MaSp sequences

In examining the overall presence and periodicity of motifs in *A. aurantia* and *L. hesperus* MaSp sequences, other striking patterns in residue composition became apparent. Specifically, each class of *A. aurantia* MaSp (MaSp1, MaSp2, or MaSp3), contained at least one sequence with an increased presence of hydrophobic (F) and charged (R, E and/or D) residues: MaSp1b, MaSp2.2a, MaSp3a and MaSp3b (Fig. 5). While *L. hesperus* MaSp1 and MaSp2 (and *A. aurantia* MaSp1s) do show an increased presence of positively charged residues (R), they lack the increased presence of hydrophobic and/or negatively charged residues seen in these unique *A. aurantia* MaSps. This suggests a potential role in the mechanical properties, and subsequently, in the different α* values of *L. hesperus* and *A. aurantia* MA fibers.

Charged and hydrophobic residues play a significant role in protein folding, through facilitating molecular interactions (Dyson et al. 2006; Tripathi et al. 2015). In addition, charged residues, if on the surfaces of spidroins, may contribute to molecular interactions in spidroin-spidroin associations within silk fibers. It is then feasible that proteins with these biochemical qualities are playing a unique role in the overall assembly of *A. aurantia* spidroins into silk fibers. The prevalence of these residues within the spidroin sequences may indicate that they are specialized for an important role in the overall structure of silk fibers.

The *A. aurantia*-specific MaSps with unique properties also seem particularly relevant to interactions with water. Positively and negatively charged residues can influence both within-protein interactions, and thus folding, as well as protein–protein associations leading to molecular assembly processes. In addition, the regular alternation of these charged residues with hydrophobic residues suggests these interactions could be relevant by participating in the reorganization of the crystalline portions, or even more generally to overall thread properties by facilitating spidroin–spidroin association.

There are disparate mechanical demands on the MA silks of *A. aurantia* and *L. hesperus*. Besides being deployed as the safety dragline, MA silk is amply used in the scaffold of *L. hesperus* cobwebs and the frame and radii of *A. aurantia* orb webs. The two types of webs capture prey differently. *L. hesperus* cobwebs capture both walking and flying prey through use of gumfoot threads, which detach from the substrate to ensnare prey (Sahni et al. 2012). MA fibers have been associated with the upper tangled portion of the cobweb, as well as the axial fibers of gumfoot lines (Blackledge et al. 2005), though recent proteomic evidence suggests that flagelliform fibers (which make up the capture spiral in orb webs) may be more present in gumfoot lines than previously thought (Ayoub et al. 2021). By contrast, spiders such as *A. aurantia* ensnare flying prey with aerial nets composed of highly extensible, sticky capture spiral silk supported by the MA fibers that form the frame and radii (Work 1981). While the capture spiral facilitates both prey retention and reduction of web damage via localized deformation (Jyoti et al. 2018, Tarakanova and Buehler 2012a, b), the radii have been found to absorb the majority (70–100%) of energy from prey impact via dissipation of forces globally (Kelly et al. 2011; Sensenig et al. 2012; Cranford et al. 2012). Other contributions to the energy absorption of prey impact have been attributed to the “secondary frame” (Soler and Zaera 2016), also composed of MA fibers. With the different modes of prey capture and tensile demands on MA silk, selection for strong but comparatively inflexible MA fibers may have been favored in cobweb spiders, while an increased raw distance of fiber extension may have been favored in orb-web weavers both to maximize the web’s ability to absorb energy from prey impact and to facilitate localized deformation within capture spiral threads for prey retention.

### Implications of MaSp molecular structure for α* parameter values

The extreme periodicity of spidroin sequences is believed to be a consequence of multiple factors, the most dramatic of which are natural selection and recombination-mediated mechanisms, such as unequal crossing over, that result in the homogenization of tandem repeats (Chaw et al. 2014; Ayoub et al. 2007, 2013; Ayoub and Hayashi 2008; Beckwitt et al. 1998). Such factors could explain the consistency of motif periodicity throughout the length of MaSp sequences. The differences in spidroin sequences of two spiders with different α* values are likely playing a role in the distinctive overall mechanical properties of these silks, such as the breaking of hydrogen bonds associated with the “entropic unfolding” deformation phase dragline fibers experience at intermediate strains (Yarger et al. 2018). It must be highlighted that the full relevance of this analysis can only be obtained after characterizing not only the differences between distinct protein sequences, but also determining their quantitative presence in the MA fibers of each species (Jorge et al. 2022). However, even without this information, it is possible to establish some key differences in the structures of *A. aurantia* and *L. hesperus* MaSps that offer clues on the origin of the different tensile properties of both materials. We found that the number of residues between poly-A motifs is higher in *A. aurantia* MaSps than in the MaSps of *L. hesperus*. This trait could allow for more amorphous, coiled regions, likely facilitating malleability of these spidroins, a quality associated with increased extensibility. In addition, in *A. aurantia* MA silk, the greater distances between poly-A motifs might be correlated with a larger hidden length, resulting from a more gradual alignment of the fiber’s semi-amorphous region in response to strain (Keten et al. 2010) compared with that spun by *L. hesperus*.

Analyses were expanded to the periodic occurrences of proline and tyrosine-associated motifs, such as GYG and GPG. Proline has been shown to be a major contributor to supercontraction (Liu et al. 2008; Savage and Gosline 2008a, b), and recent analyses of synthetic MA fibers have demonstrated that tyrosine (Y) residues may also be playing an integral role (Greco et al. 2021). Due to the variability of observed Y-associated motifs (GYG; all MaSps, GRYG: *A. aurantia* MaSp1a, MaSp3a, MaSp3b; PYG: *A. aurantia* MaSp2.1a, MaSp2.2a, MaSp2.2b, MaSp2.2c, MaSp2.2d, MaSp2.2e; SY: *A. aurantia* MaSp2.1b; VY: *A. aurantia* MaSp2.1b; AY: *A. aurantia* MaSp2.2e, *L. hesperus* MaSp2), analysis was expanded to the higher level periodicity of the tyrosine residue itself to better investigate the relationship between tyrosine and supercontraction potential. Tyrosine residues were found to occur with conserved, higher level periodicities in all MaSp sequences of both species, typically ~ 5–40 amino acids apart (Supplemental Fig. 4), though a single case, A. *aurantia* MaSp2.2a, showed larger distances between tyrosine residues overall (60–100 aa). This suggests that tyrosine’s periodic occurrence within MaSps may be a basic property of dragline fibers, regardless of the involved motif, adding weight to tyrosine’s proposed importance in supercontraction (Greco et al. 2021). Within *A. aurantia* MaSp1s, the average distances between tyrosine residues varied (27.5 MaSp1a; 63.5 MaSp1b), while the average distance between tyrosine residues of *L. hesperus* MaSp1 was smaller (19.2). This value observed in *L. hesperus* MaSp1 was strikingly similar to those seen in all other MaSps, regardless of MaSp class or species (mean: 17.5, range 17.3–21.7), with the exception of *A. aurantia* MaSp2.2a (64.5) which showed values similar to *A. aurantia* MaSp1b.

Proline-containing motifs were also examined, through the analysis of the periodic occurrences of proline (P) residues. *L. hesperus* MaSp2 showed relatively little obvious periodicity, while conservation of P periodic occurrence was well observed in *A. aurantia* MaSp2s, particularly within the MaSp2.2s. Within all MaSp2 sequences, P distances ranged from 2 to 15 amino acids (Supplemental Fig. 5), with comparatively shorter distances in *A. aurantia* MaSp2s (mean: 6.6 vs 10.4 in *L. hesperus*). This depicts a clear difference between MaSp2 sequences of *L. hesperus* and *A. aurantia*. Taken together with the results of tyrosine periodicity, it appears that the presence and occurrence of tyrosine residues may provide base-level supercontraction potential to all MaSps, but that this supercontraction potential is improved upon by the presence and periodic occurrences of proline residues. The presence of tyrosine-associated motifs, like GGY (Malay et al. 2017), then, may explain the ability of RTA spider MaSps to supercontract, though only about half as well as those of araneoid spiders, despite lacking proline-containing MaSp2s (Pérez-Rigueiro et al. 2010; Boutry and Blackledge 2010).

## Conclusion

This study highlights several qualities of MaSp sequences that may be influencing the overall structural and mechanical properties of MA fibers in the context of the α* parameter, such as the presence/absence of the SQ/QQ motifs, increased presence of the GLG motif type in *A. aurantia* MaSp1s, the absence of the GYG motif in *A. aurantia* MaSp2.2a and the absence of poly-GA motifs in most *A. aurantia* MaSp2 sequences. The presence of spidroins with polar, charged, and hydrophobic residues is also likely to impact the folding and/or assembly of the spidroins through various molecular interactions, such as hydrogen bonds, and to influence the interaction of the proteins with water. These interactions might be relevant both for the spinning of the fiber from the viscous silk dope and for the performance of the solid material. Supercontraction of spider silks is, in part, associated with the ability of the crystalline portions of silks to change their orientation in response to water, a process which could be facilitated by such interactions. In addition, the periodicity of some residues, like tyrosine, within the sequences is strongly conserved. This conservation of distance suggests that they may be facilitating important, and identifiable interactions during the assembly of spidroins into silk fibers and are likely to be influencing the overall properties of dragline silks.

Overall motif composition (particularly GXG) and increased glycine-rich region length may contribute to define the hidden length exhibited by different proteins and, consequently, be relevant to silk fiber mechanical properties. Periodicity is also likely to play a role here, as we see distinct differences between *A. aurantia* and *L. hesperus*, with *A. aurantia* showing more conservation of periodicity in MaSp2 sequences, while *L. hesperus* exhibiting more periodicity in MaSp1. In addition, while periodic occurrence of tyrosine residues is shown to be a base-level characteristic of all spidroins examined, we did find higher level periodic occurrence of proline in *A. aurantia* MaSp2s that is absent in the MaSp2 of *L. hesperus*, implicating proline as a contributor to variance in α* values. Our study also suggests that the overall sequence coverage and uninterrupted length of motifs are unlikely to play a critical role in the structural and mechanical properties of dragline silks.

This study of two exemplar spiders should be expanded to additional species to test hypotheses on the relevance of these molecular components in overall fiber tensile properties, as α* values and full-length spidroin sequences become available. We cannot at present discount the potential role of other factors, such as post-translational modifications or overall number of MaSp genes, in supercontraction potential of MaSp fibers. For example, expression levels of the different MaSps in the major ampullate gland are needed to fully contextualize the sequence comparisons and proteomic analysis is needed to confirm the presence of MaSps in dragline fibers. While both MaSp1 and Masp2 are expressed within the dragline fibers of *L. hesperus* (Chaw et al. 2015), no proteomic studies have been conducted for *A. aurantia*. Similarly, the architecture of the fibers themselves, including microstructures such as nonfibrillar bundles (Wang and Schniepp 2018; Giesa et al. 2011), may be impacting the overall mechanical properties of silk fibers. Such architectural qualities would be subject not only to the molecular properties of the spidroins forming the fibers, but also to the physical constraints of spider silk-spinning systems (Knight and Vollrath 2001, 2002; Chen et al. 2006). These are, however, significant endeavors requiring combined genomic and proteomic analyses, the first of which (in the spider *Trichonephila clavipes*) has just recently been published (Jorge et al. 2022). The present study, however, offers a primer on identifying the relevant molecular differences between spidroins that substantiate their wide range of mechanical properties.

## Supplementary Information

Below is the link to the electronic supplementary material.

Supplemental Table 1 Overlap of GGX and GXG Motifs. Matrices depicting overlap of GGX and GXG motifs within analyzed MaSps. Colored boxes denote MaSp type (blue, MaSp1; green, MaSp2; orange, MaSp3) and total number of motif occurrences in full-length MaSp sequences. Boxes contain the total number of overlapping residues between motifs, where yellow boxes denote non-zero values. Grey boxes highlight instances of GGX/GXG motif overlap (PDF 247 kb)

Supplemental Fig. 1 Schematic of method for quantifying uninterrupted length of consecutively occurring motifs. Each spidroin repetitive region was scanned for a particular motif, such as GXG (left) and concurrent motifs were extracted (middle). Unless interrupted by another residue, lengths of the concurrent motifs were recorded and then mean motif size was calculated (right). Runs of concurrent motifs included both cases in which motifs overlapped (underlined), and in which one motif immediately followed another (yellow) (PDF 37 kb)

Supplemental Fig. 2 Individual Motif Percentage of Major Ampullate Spidroins. Quantification of overall motif coverage in full-length MaSp sequences of *A. aurantia* and *L. hesperus*. GGX and GXG motifs portions are displayed as individual motif types. Percentages calculated after correction for overlapping residues (PDF 42 kb)

Supplemental Fig. 3 Periodicity of Glycine-Rich Region Length in MaSp2s. Glycine-rich regions of all *Argiope aurantia* and *Latrodectus hesperus* full-length MaSp2 sequences in order of occurrence. Points represent the length (in residues, y-axis) and start position (x-axis) of an individual region (PDF 111 kb)

Supplemental Fig. 4 Periodicity of Tyrosine (Y) Residues. Details of tyrosine periodicity in the repetitive region of all examined *Argiope aurantia* and *Latrodectus hesperus* MaSp sequences. The Y axis denotes the distances (in amino acids) between proline residues and the X axis denotes the proline residue’s position in the sequence (PDF 214 kb)

Supplemental Fig. 5 Periodicity of Proline Residues. Details of proline periodicity in repetitive the region of all examined *Argiope aurantia* and *Latrodectus hesperus* MaSp2 sequences. The Y axis denotes the distances (in amino acids) between proline residues and the X axis denotes the proline residue’s position in the sequence (PDF 263 kb)

Supplemental Fig. 6 Full-Length Motif Visualization of MaSps. All full-length MaSp sequences, color-coded for motifs (as in Figure 1) associated with fiber mechanical properties in literature (PDF 413 kb)
