# Supplementary material for: Relating spidroin motif prevalence and periodicity to the mechanical properties of major ampullate spider silks
Source: J Comp Physiol B. 2022 Nov 7;193(1):25–36. doi: 10.1007/s00360-022-01464-3 (PMC9852138; doi:10.1007/s00360-022-01464-3)
Supplement: Supplementary file 1 — Supplemental Table 1 Overlap of GGX and GXG Motifs. Matrices depicting overlap of GGX and GXG motifs within analyzed MaSps. Colored boxes denote MaSp type (blue, MaSp1; green, MaSp2; orange, MaSp3) and total number of motif occurrences in full-length MaSp sequences. Boxes contain the total number of overlapping residues between motifs, where yellow boxes denote non-zero values. Grey boxes highlight instances of GGX/GXG motif overlap (PDF 247 kb) [file 360_2022_1464_MOESM1_ESM.pdf]

A. aurantia

MaSp1a	GGA	GGL	GGQ	GGR	GGY	GLG	GRG	GYG	GQG	GPG
	194	78	123	0	78	111	45	104	246	1
**GGA**	X	0	0	0	0	0	0	0	94	0
**GGL**	0	X	0	0	0	156	32	45	1	0
**GGQ**	0	0	X	0	0	33	0	0	283	0
**GGR**	0	0	0	X	0	0	0	0	0	0
**GGY**	0	0	0	0	X	0	0	156	76	1
**GAG**	x	x	x	x	x	x	x	x	x	x
**GLG**	0	156	33	0	0	X	0	0	0	0
**GRG**	0	32	0	0	0	0	X	0	0	0
**GYG**	0	45	0	0	156	0	0	X	26	0
**GQG**	94	1	283	0	76	0	0	26	X	0
**GPG**	0	0	0	0	1	0	0	0	0	X

MaSp1b	GGA	GGL	GGQ	GGR	GGY	GLG	GRG	GYG	GQG	GPG
	178	88	1	44	1	182	45	51	89	1
**GGA**	X	0	0	0	0	47	0	0	27	1
**GGL**	0	X	0	0	0	174	0	0	0	0
**GGQ**	0	0	X	0	0	0	0	0	0	0
**GGR**	0	0	0	X	0	41	88	0	0	0
**GGY**	0	0	0	0	X	0	0	0	0	0
**GAG**	x	x	x	x	x	x	x	x	x	x

GLG	47	174	0	41	0	X	0	0	0	0
GRG	0	0	0	88	0	0	X	0	0	0
GYG	0	0	0	0	0	0	0	X	46	0
GQG	27	0	0	0	0	0	0	46	X	0
GPG	1	0	0	0	0	0	0	0	0	X

MaSp2.1a	GGA	GGL	GGQ	GGR	GGY	GLG	GRG	GYG	GQG	GPG
	20	1	71	1	50	0	5	52	87	164
GGA	X	0	0	0	0	0	0	0	0	17
GGL	0	X	0	0	0	0	0	0	0	0
GGQ	0	0	X	0	0	0	0	0	153	29
GGR	0	0	0	X	0	0	2	0	0	1
GGY	0	0	0	0	X	0	4	100	0	45
GAG	x	x	x	x	x	x	x	x	x	x
GLG	0	0	0	0	0	X	0	0	0	0
GRG	0	0	0	2	4	0	X	0	4	0
GYG	0	0	0	0	100	0	0	X	0	0
GQG	0	0	153	0	0	0	4	0	X	8
GPG	17	0	29	1	45	0	0	0	8	X

MaSp2.1b	GGA	GGL	GGQ	GGR	GGY	GLG	GRG	GYG	GQG	GPG
	77	1	3	0	120	3	1	136	4	338
GGA	X	0	0	0	0	0	0	0	0	75
GGL	0	x	0	0	0	0	0	0	0	0

**GGQ**	0	0	x	0	0	0	0	0	6	2
**GGR**	0	0	0	x	0	0	0	0	0	0
**GGY**	0	0	0	0	x	2	0	240	0	67
**GAG**	x	x	x	x	x	x	x	x	x	x
**GLG**	0	0	0	0	2	x	0	0	0	0
**GRG**	0	0	0	0	0	0	x	1	0	0
**GYG**	0	0	0	0	240	0	1	x	1	120
**GQG**	0	0	6	0	0	0	0	1	x	1
**GPG**	75	0	2	0	67	0	0	120	1	x

**MaSp2.2a**	**GGA**	**GGL**	**GGQ**	**GGR**	**GGY**	**GLG**	**GRG**	**GYG**	**GQG**	**GPG**
	87	1	113	1	4	0	0	4	48	302
**GGA**	x	0	0	0	0	0	0	0	1	73
**GGL**	0	x	0	0	0	0	0	0	0	0
**GGQ**	0	0	x	0	0	0	0	0	90	44
**GGR**	0	0	0	x	0	0	0	0	0	0
**GGY**	0	0	0	0	x	0	0	8	1	0
**GAG**	x	x	x	x	x	x	x	x	x	x
**GLG**	0	0	0	0	0	x	0	0	0	0
**GRG**	0	0	0	0	0	0	x	0	0	0
**GYG**	0	0	0	0	8	0	0	x	1	3
**GQG**	1	0	90	0	1	0	0	1	x	0
**GPG**	73	0	44	0	0	0	0	3	0	x

MaSp2.2b	GGA	GGL	GGQ	GGR	GGY	GLG	GRG	GYG	GQG	GPG
	72	1	136	0	72	1	4	74	90	374
GGA	x	0	0	0	0	0	0	0	0	71
GGL	0	x	0	0	0	0	0	0	0	0
GGQ	0	0	x	0	0	0	0	0	172	86
GGR	0	0	0	x	0	0	0	0	0	0
GGY	0	0	0	0	x	0	0	144	0	0
GAG	x	x	x	x	x	x	x	x	x	x
GLG	0	0	0	0	0	x	0	0	0	0
GRG	0	0	0	0	0	0	x	0	0	0
GYG	0	0	0	0	144	0	0	x	2	72
GQG	0	0	172	0	0	0	0	2	x	0
GPG	71	0	86	0	0	0	0	72	0	x

MaSp2.2c	GGA	GGL	GGQ	GGR	GGY	GLG	GRG	GYG	GQG	GPG
	48	1	133	0	71	0	0	71	87	326
GGA	x	0	0	0	0	0	0	0	0	48
GGL	0	x	0	0	0	0	0	0	0	0
GGQ	0	0	x	0	0	0	0	0	168	80
GGR	0	0	0	x	0	0	0	0	0	0
GGY	0	0	0	0	x	0	0	142	1	0
GAG	x	x	x	x	x	x	x	x	x	x
GLG	0	0	0	0	0	x	0	0	0	0

GRG	0	0	0	0	0	0	x	0	0	0
GYG	0	0	0	0	142	0	0	x	1	64
GQG	0	0	168	0	1	0	0	1	x	0
GPG	48	0	80	0	0	0	0	64	0	x

MaSp2.2d	GGA	GGL	GGQ	GGR	GGY	GLG	GRG	GYG	GQG	GPG
	47	1	134	0	70	0	1	70	86	337
GGA	x	0	0	0	0	0	0	0	0	47
GGL	0	x	0	0	0	0	0	0	0	0
GGQ	0	0	x	0	0	0	1	0	166	83
GGR	0	0	0	x	0	0	0	0	0	0
GGY	0	0	0	0	x	0	0	140	1	0
GAG	x	x	x	x	x	x	x	x	x	x
GLG	0	0	0	0	0	x	0	0	0	0
GRG	0	0	1	0	0	0	x	0	0	1
GYG	0	0	0	0	140	0	0	x	1	69
GQG	0	0	166	0	1	0	0	1	x	0
GPG	47	0	83	0	0	0	1	69	0	x

MaSp2.2e	GGA	GGL	GGQ	GGR	GGY	GLG	GRG	GYG	GQG	GPG
	28	1	80	1	48	0	0	50	55	220
GGA	x	0	0	0	0	0	0	0	0	28
GGL	0	x	0	0	0	0	0	0	0	0
GGQ	0	0	x	0	0	0	0	0	104	44

**GGR**	0	0	0	x	0	0	0	0	0	0
**GGY**	0	0	0	0	x	0	0	96	1	0
**GAG**	x	x	x	x	x	x	x	x	x	x
**GLG**	0	0	0	0	0	x	0	0	0	0
**GRG**	0	0	0	0	0	0	x	0	0	0
**GYG**	0	0	0	0	96	0	0	x	1	49
**GQG**	0	0	104	0	1	0	0	1	x	1
**GPG**	28	0	44	0	0	0	0	49	1	x

**MaSp3a**	**GGA**	**GGL**	**GGQ**	**GGR**	**GGY**	**GLG**	**GRG**	**GYG**	**GQG**	**GPG**
	47	3	13	42	28	20	24	69	40	65
**GGA**	x	0	0	0	0	11	1	0	2	20
**GGL**	0	x	0	0	0	0	0	0	0	2
**GGQ**	0	0	x	0	0	0	0	4	20	0
**GGR**	0	0	0	x	0	0	47	0	2	0
**GGY**	0	0	0	0	x	0	0	56	0	0
**GAG**	x	x	x	x	x	x	x	x	x	x
**GLG**	11	0	0	0	0	x	0	0	0	1
**GRG**	1	0	0	47	0	0	x	1	0	0
**GYG**	0	0	4	0	56	0	1	x	19	4
**GQG**	2	0	20	2	0	0	0	19	x	0
**GPG**	20	2	0	0	0	1	0	4	0	x

**MaSp3b**	**GGA**	**GGL**	**GGQ**	**GGR**	**GGY**	**GLG**	**GRG**	**GYG**	**GQG**	**GPG**

	69	2	18	51	24	2	37	73	58	55
**GGA**	x	0	0	0	0	1	0	0	1	39
**GGL**	0	x	0	0	0	0	0	0	0	1
**GGQ**	0	0	x	0	0	0	0	16	34	0
**GGR**	0	0	0	x	0	0	71	0	16	0
**GGY**	0	0	0	0	x	1	0	48	2	0
**GAG**	x	x	x	x	x	x	x	x	x	x
**GLG**	1	0	0	0	1	x	0	0	0	0
**GRG**	0	0	0	71	0	0	x	0	0	0
**GYG**	0	0	16	0	48	0	0	x	16	0
**GQG**	1	0	34	16	2	0	0	16	x	0
**GPG**	39	1	0	0	0	0	0	0	0	x

L. hesperus

**MaSp1**	**GGA**	**GGL**	**GGQ**	**GGR**	**GGY**	**GLG**	**GRG**	**GYG**	**GQG**	**GPG**
	227	0	70	1	142	0	43	142	330	1
**GGA**	x	0	0	0	0	0	21	0	134	0
**GGL**	0	x	0	0	0	0	0	0	0	0
**GGQ**	0	0	x	0	0	0	0	21	186	0
**GGR**	0	0	0	x	0	0	1	0	0	0
**GGY**	0	0	0	0	x	0	19	283	119	0
**GAG**	x	x	x	x	x	x	x	x	x	x

GLG	0	0	0	0	0	x	0	0	0	0
GRG	21	0	0	1	19	0	x	38	0	0
GYG	0	0	21	0	283	0	38	x	78	0
GQG	134	0	186	0	119	0	0	78	x	0
GPG	0	0	0	0	0	0	0	0	0	x

MaSp2	GGA	GGL	GGQ	GGR	GGY	GLG	GRG	GYG	GQG	GPG
	95	0	40	1	13	1	0	139	24	239
GGA	x	0	0	0	0	0	0	0	0	43
GGL	0	x	0	0	0	0	0	0	0	0
GGQ	0	0	x	0	0	0	0	28	2	9
GGR	0	0	0	x	0	0	0	0	0	0
GGY	0	0	0	0	x	0	0	26	0	12
GAG	x	x	x	x	x	x	x	x	x	x
GLG	0	0	0	0	0	x	0	1	0	0
GRG	0	0	0	0	0	0	x	0	0	0
GYG	0	0	28	0	26	1	0	x	18	104
GQG	0	0	2	0	0	0	0	18	x	0
GPG	43	0	9	0	12	0	0	104	0	x
