# Supplementary material for: Relating spidroin motif prevalence and periodicity to the mechanical properties of major ampullate spider silks
Source: J Comp Physiol B. 2022 Nov 7;193(1):25–36. doi: 10.1007/s00360-022-01464-3 (PMC9852138; doi:10.1007/s00360-022-01464-3)
Supplement: Supplementary file 2 — Supplemental Fig. 1 Schematic of method for quantifying uninterrupted length of consecutively occurring motifs. Each spidroin repetitive region was scanned for a particular motif, such as GXG (left) and concurrent motifs were extracted (middle). Unless interrupted by another residue, lengths of the concurrent motifs were recorded and then mean motif size was calculated (right). Runs of concurrent motifs included both cases in which motifs overlapped (underlined), and in which one motif immediately followed another (yellow) (PDF 37 kb) [file 360_2022_1464_MOESM2_ESM.pdf]

Spidroin Repetitive Sequence

AAAAAAAAGGAGGAGRGGLG
AGGAGQGYGSGLGGQGGAGQ
GGAAAAAAAAGGQGGQGGYG
GLGSQGAGQGGAGAAAAAAA
AGGAGGAGRGGLGAGGAGQG
YGSGLGGQGGAGGGAAAAAA
AAGGQGGQGGYGGLGSQGAS

Contiguous Motifs (Ordered by Length)

GQG GQG

GQGYG GQGYG

GRGGLG GLGGQG
GRGGLG GLGGQG

GQGGQGGYGGLG
GQGGQGGYGGLG

Mean Motif Size

6.4 aa
