# Supplementary figures and images for: Relating spidroin motif prevalence and periodicity to the mechanical properties of major ampullate spider silks
Source: J Comp Physiol B. 2022 Nov 7;193(1):25–36. doi: 10.1007/s00360-022-01464-3 (PMC9852138; doi:10.1007/s00360-022-01464-3)

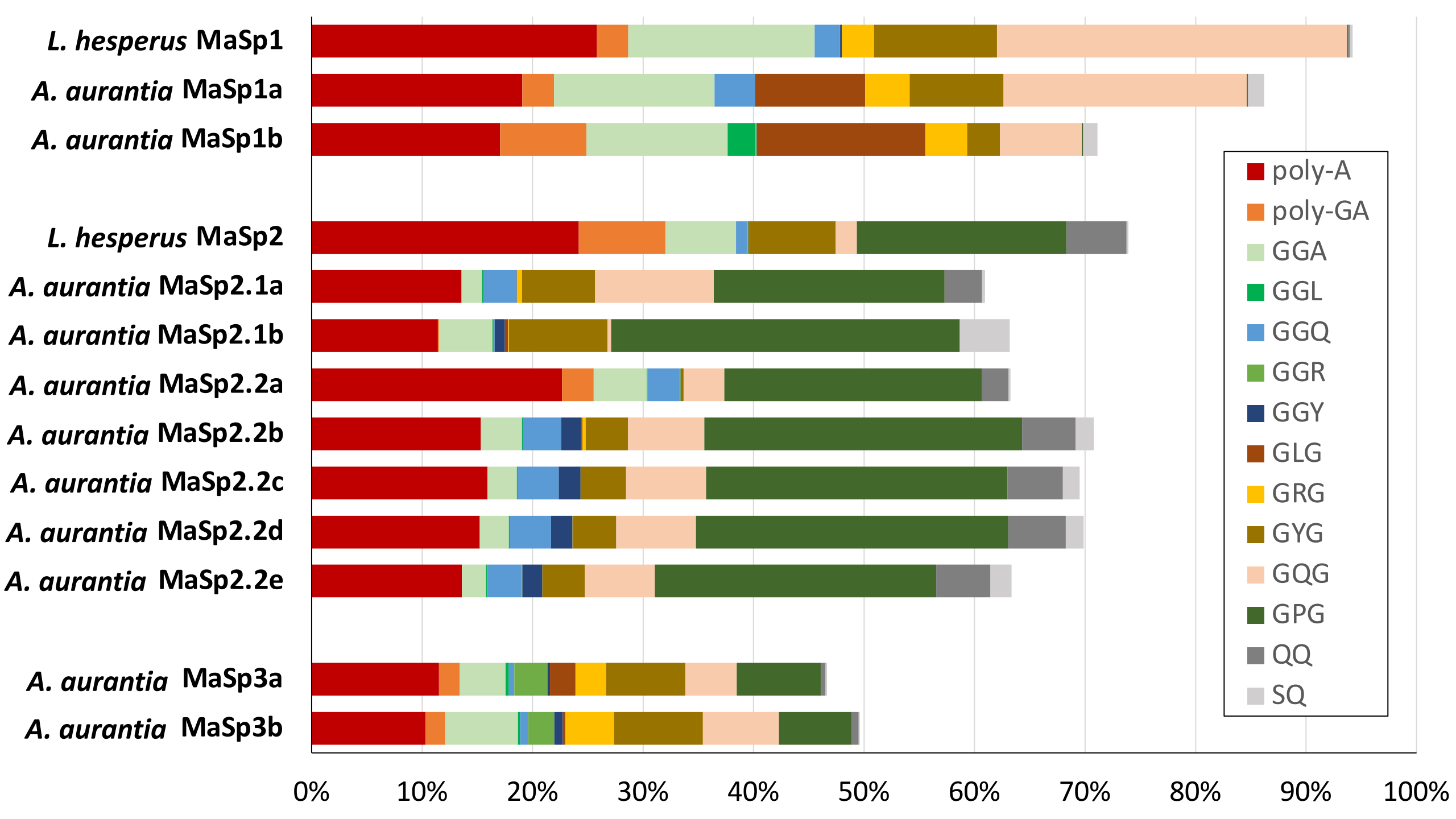

Supplement: Supplementary file 3 — Supplemental Fig. 2 Individual Motif Percentage of Major Ampullate Spidroins. Quantification of overall motif coverage in full-length MaSp sequences of A. aurantia and L. hesperus. GGX and GXG motifs portions are displayed as individual motif types. Percentages calculated after correction for overlapping residues (PDF 42 kb) [file 360_2022_1464_MOESM3_ESM.pdf]

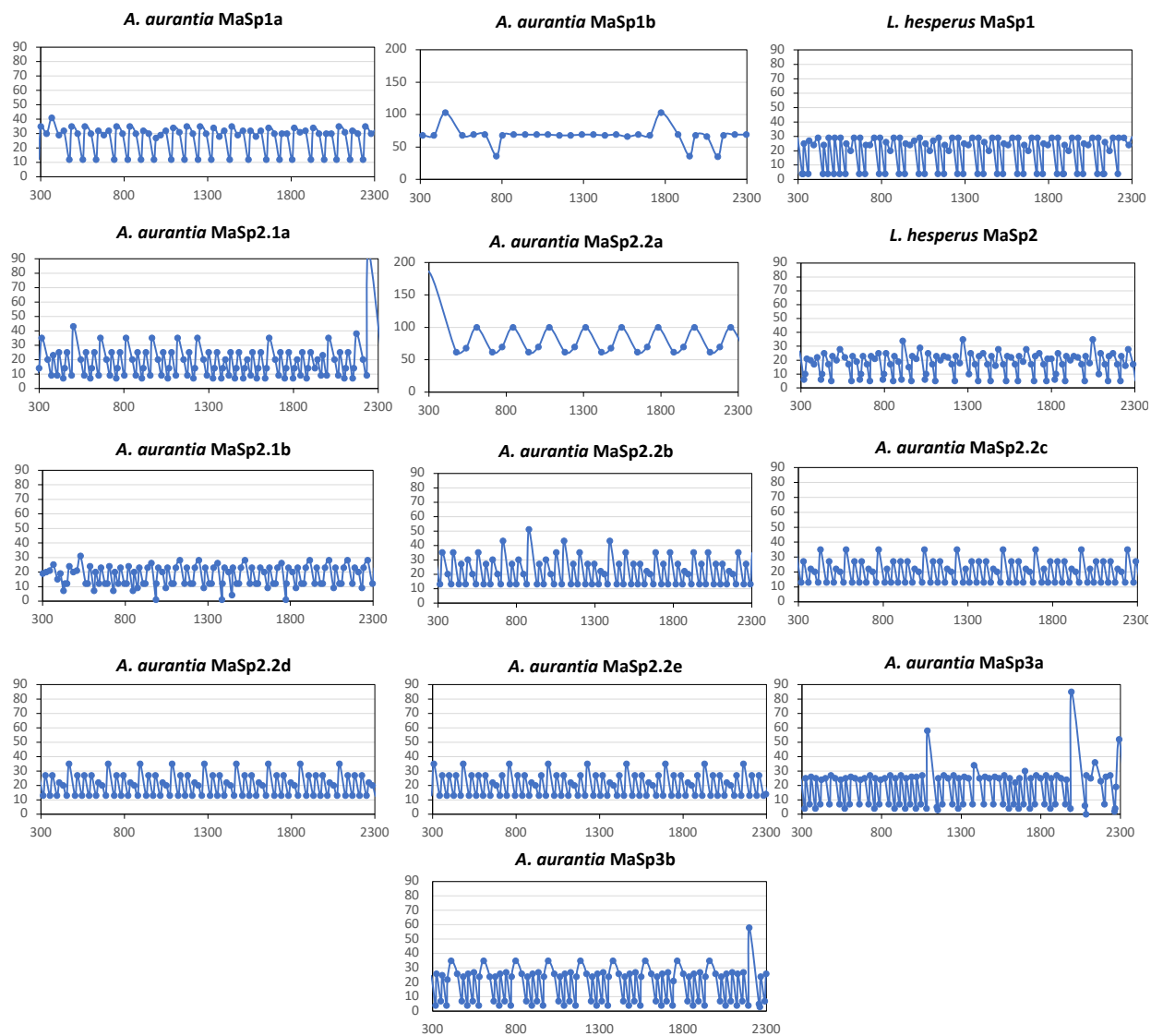

Tyrosine

Supplement: Supplementary file 5 — Supplemental Fig. 4 Periodicity of Tyrosine (Y) Residues. Details of tyrosine periodicity in the repetitive region of all examined Argiope aurantia and Latrodectus hesperus MaSp sequences. The Y axis denotes the distances (in amino acids) between proline residues and the X axis denotes the proline residue’s position in the sequence (PDF 214 kb) [file 360_2022_1464_MOESM5_ESM.pdf]
