# Supplementary material for: Relating spidroin motif prevalence and periodicity to the mechanical properties of major ampullate spider silks
Source: J Comp Physiol B. 2022 Nov 7;193(1):25–36. doi: 10.1007/s00360-022-01464-3 (PMC9852138; doi:10.1007/s00360-022-01464-3)
Supplement: Supplementary file 4 — Supplemental Fig. 3 Periodicity of Glycine-Rich Region Length in MaSp2s. Glycine-rich regions of all Argiope aurantia and Latrodectus hesperus full-length MaSp2 sequences in order of occurrence. Points represent the length (in residues, y-axis) and start position (x-axis) of an individual region (PDF 111 kb) [file 360_2022_1464_MOESM4_ESM.pdf]

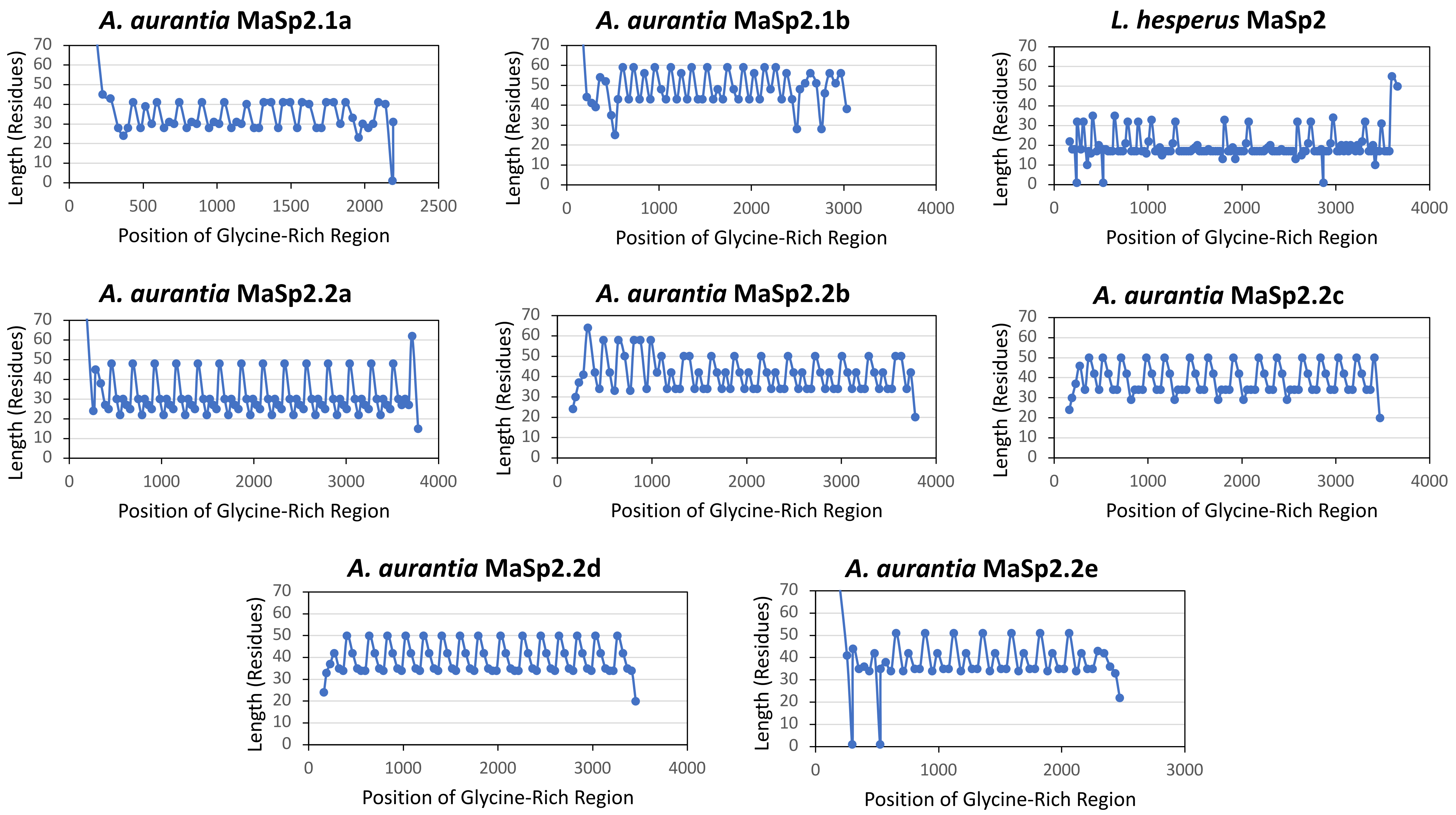
A. aurantia MaSp2.1a
A. aurantia MaSp2.1b
L. hesperus MaSp2
A. aurantia MaSp2.2a
A. aurantia MaSp2.2b
A. aurantia MaSp2.2c
A. aurantia MaSp2.2d
A. aurantia MaSp2.2e
Length (Residues)
Position of Glycine-Rich Region
0
10
20
30
40
50
60
70
500
1000
1500
2000
2500
3000
4000
