# Supplementary material for: Relating spidroin motif prevalence and periodicity to the mechanical properties of major ampullate spider silks
Source: J Comp Physiol B. 2022 Nov 7;193(1):25–36. doi: 10.1007/s00360-022-01464-3 (PMC9852138; doi:10.1007/s00360-022-01464-3)
Supplement: Supplementary file 6 — Supplemental Fig. 5 Periodicity of Proline Residues. Details of proline periodicity in repetitive the region of all examined Argiope aurantia and Latrodectus hesperus MaSp2 sequences. The Y axis denotes the distances (in amino acids) between proline residues and the X axis denotes the proline residue’s position in the sequence (PDF 263 kb) [file 360_2022_1464_MOESM6_ESM.pdf]

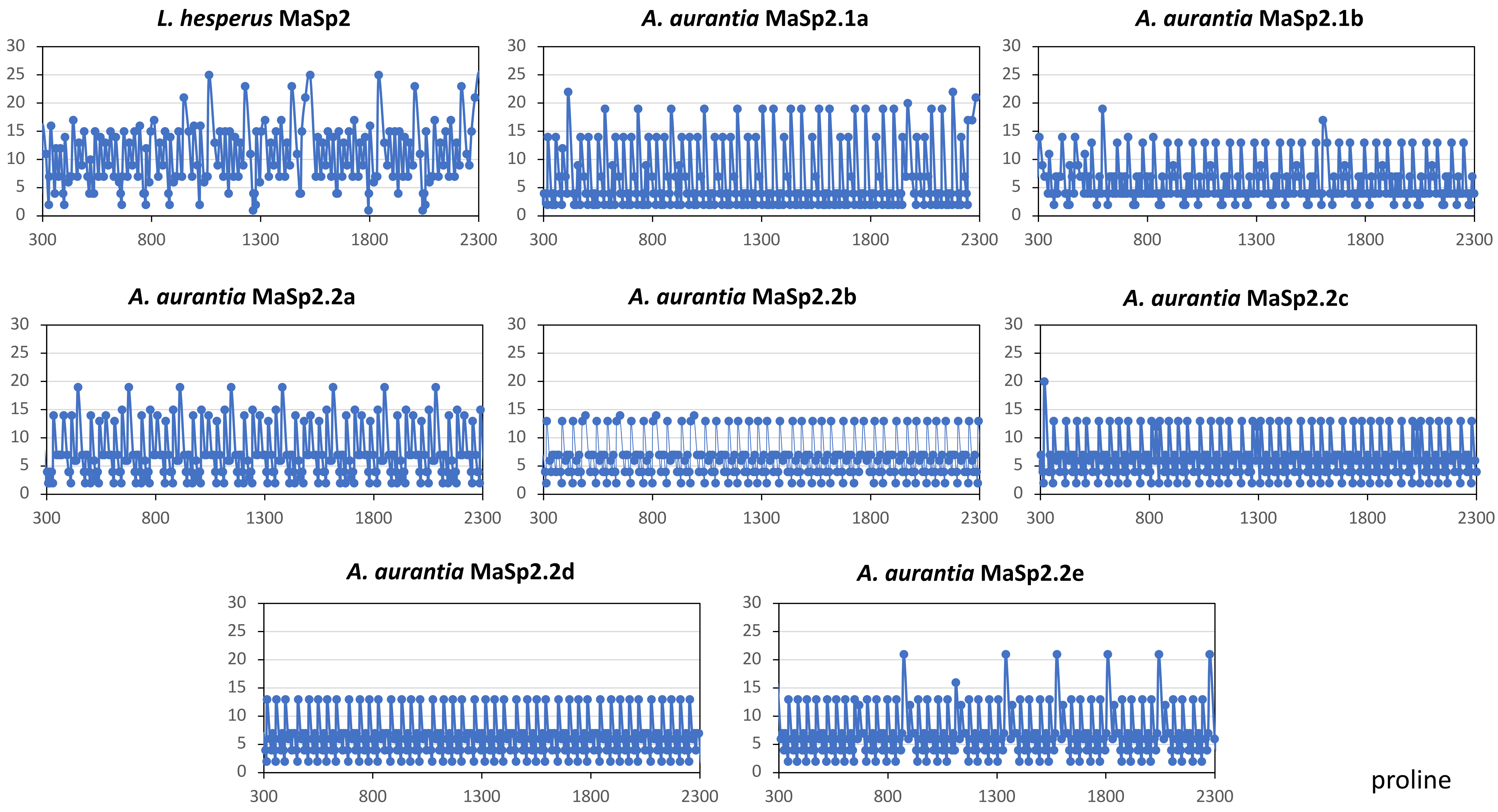

L. hesperus MaSp2
A. aurantia MaSp2.1a
A. aurantia MaSp2.1b
A. aurantia MaSp2.2a
A. aurantia MaSp2.2b
A. aurantia MaSp2.2c
A. aurantia MaSp2.2d
A. aurantia MaSp2.2e
0 5 10 15 20 25 30
300 800 1300 1800 2300
proline
