# Supplementary material for: Relating spidroin motif prevalence and periodicity to the mechanical properties of major ampullate spider silks
Source: J Comp Physiol B. 2022 Nov 7;193(1):25–36. doi: 10.1007/s00360-022-01464-3 (PMC9852138; doi:10.1007/s00360-022-01464-3)
Supplement: Supplementary file 7 — Supplemental Fig. 6 Full-Length Motif Visualization of MaSps. All full-length MaSp sequences, color-coded for motifs (as in Figure 1) associated with fiber mechanical properties in literature (PDF 413 kb) [file 360_2022_1464_MOESM7_ESM.pdf]

>Aaur_Masp1a
MNWTTRLALSVLVVICSQSIFALGQSPWQSASMAESFMTYFSAALGQSGAFTNEQMDDIDTIATSIKMGVD
KMERSGKTSQNKLQAMNMAFASAVAEIAIAEGGGQSAQVKTNAIADALASAFLQTTGVVNRQFINEIRGLIS
MFAQANSISSSSGYASASAEAAAGSAGGAGQGYGAGLGGQGGAGQGGAAAAAAAAGGQGGQGGYGGL
GSQGAGQGGYGAGQGGAGAAAAAAAGGAGGAGRGGLGAGGGGQGYGSGLGGQGGAGQGGAAAA
AAAAGGQGGQGGYGGLGSQGAGQGGYGAGQGGAGAAAAAAAGGAGGAGRGGLGAGGAGQGYGS
GLGGQGGAGQGGAAAAAAAAGGQGGQGGYGGLGSQGAGQGGAGAAAAAAAGGAGGAGRGGLGA
GGAGQGYGSGLGGQGGAGGGAAAAAAAAGGQGGQGGYGGLGSQGASQGGAGRGAAAAAAAAGGQG
GQGGYGGLGSQGAGQGGYGAGQGGAGAAAAAAAGGTGGAGRGGLGAGGAGQGYGSGLGGQGGAG
QGGAAAAAAAAGGQGGLGGYGGLGSQGAGQGGYGAGQGGAGAAAAAAAGGAGGAGRGGLGAGGA
GQGYGSGLGGQGGAGQGGAAAAAAAAGSQGGQGGYGGLGSQGAGQGGYGAGQGGAGAAAAAAA
GGAGRGGLGAGGAGQGYGSGLGGQGGAGGGAAAAAAAAGGQGGQGGYGGLGSQSAGQVGAGRGAA
AAAAAAGGQGGQGGYGGLGSQGAGQGGYGAGQGGAGAAAAAAAGGAGGAGRGGLGAGGAGQGY
GSGLGGQGGAGQGGAAAAAAAAGGQGGQGGYGGLGSQGAGQGGYGAGQRGAGAAAAAAAGGAG
GAGRGGLGAGGAGQGYGSGLGGQGGAGQGGAAAAAAAAGDQGGQGGYGGLGSQGAGPGGYGAGQG
GAGAAAAAAAGGAGRGGLGAGGAGQGYGSGLGGQGGAGQGGAAAAAAASGGQGGQGGYGGLGSQ
GAGQGGYGAGAAAAAAAGGAGRGGLGAGGAGQGYGSGLGGQGGARGGAAAAAAAASGQGGQGGY
GGLGSQGAGQGGAGRGASAAAAAAGGQGGQGGYGGLGSQGAGQGGYGAGQGGAGAAAAAADGGS
GGAGRGGLGAGGAGRYGSGLGGQGGDGQGGAAAAAAAAAGGQSGQGGYGGLGSQGAGQGGYGAGQ
GGAGAAAAAAAGGAGGAGRGGLGAGGAGQGYGSRLGGQGRAGQGGAAAAAAAAGGQGGQGGYGG
LGSQGAGQGGYGAGQGGAGAAAAAAAGAAGGAGRGGLGAGGAGQGYGSGLGGQGGAGQGGAAAA
AAAAGGQGGQGGYGGLGSQGAGQGGYGAGQGGAGAAAAAAAGGAGGAGRGGLGAGGAGRYGSGL
GGQGGDGQGGAAAAAAAAAGGQGGYGGLGSQGAGQGGAGRGAAAAAAAAGGQGGQGGYGGLGSQ
GAGQGGYGAGQGGAGAAAAAAAGGAGGAGRGGLGAGGAGQGYGSGLGGQGGAGGGAAAAAAAA
GGQGGQGGYGGLGSQGAGQGGAGRGASAAAAAAGGQGGQGGYGRLGSQGAGQGGYGAGQGGAGA
AAAAAGGAGGAGRGGLGAGGAGRYGSGLGGQGGDGQGGAAAAAAAAAGGQGGYGGLGSQGVGQGG
AGRGAAAAAAAAGGQGGQGGYGGLGSQGAGQGGYGAGQGGAGAAAAAAAGGAGGAGRGGLGAG
GAGRYGSGLGGQGGAGQGGAAAAAAAAGGQGGQGGYGGLGSQGAGQGGYGAGAAAAAAAGGAGG
AGRGGLGAGGAGQGYGSGLGGQGGAGQGGAAAAAAAAGGQGGQGGYGGLGSQGAGQGGYGAGQG
GAGAAAAAAAGGAGGAGRGGLGAGGAGRYGSGLGGQGGDGQGGAAAAAAAAAGGQGGQGGYGGL
GSQGAGQGGAGRGAAAAAAAAGGQGGQGGYGGLGSQGAGQGGYGAGQGGAGAAAAAAAGGAGG
AGRGGIGAGGAGRYGSGLGGQGGAGQGGAAAAAAAAGGQGGQGGYGGLGSQGAGQGGYGAGAAA
AAAAGGAGGAGRGGLGAGGAGQGYGSGLGGQGGAGQGGAAAAAAAAGGQGGQGGYGGLGSQGAG
QGGYGAGQGGAGAAAAAAAGGAGGAGRGGLGAGGAGQGYGSGLGGQGGAGQGGAAAAAAAAAG
GQGGQGGYGGLGSQGAGQGGYGAGQGGAGAAAAGGAGGAGRGGLGAGGAGQGYGSGLGGQGGA
GQGGAAAAAAAAGGQGGQGGYGGLGSEGAGQGGYGAGQGGAGAAAAAAAGGAGGAGRGGLGAG
GAGQGYGSGLGGQGGAGQGGAAAAAAAAGGQGGQGGYGGLGSQGAGQGGAGRGAAAAAAAAGGQ
GGQGGYGGLGSQGAGQGGYGAGQGGAGAAAAAAAGGAGGAGRGGLGAGGAGQGYGSGLGGQGG
AGQGGAAAAAAAAGGQGGQGGYGGLGSQGAGQGGYGAGQGGAGAAAAATAGGAGGAGRGGLGA
GGAGQGYGSGLGGQGGAGQGGAAAAAAAAGGQGGQGGYGGLGSQGAGQGGYGAGAAAAAAAGG
AGGAGRGGLGAGGAGQGYGSGLGGQGGAGQGGAAAAAAAAGGQGGQGGYGGLGSQGAGQGGYGA
GQGGAGAAAAAAAGGAGGAGRGGLGAGGAGRYGSGLGGQGGDGQGGAAAAAAAAAGGQGGQGG
YGGLGSQGVGRDAAAAAAAAGGQGGQGGYGGLGSQGAGQGGYGAGQGGAGAAAAAAAGGAGGAG
RGGLGAGGAGQGYGSGLGGQGGAGQGGAAAAAAAAAGGQGGQGGYGGLGSQGAGQGGYGVGQRG

AGAAAAAAAAGGAGGAGRGGLGAGGAGQVYGSGLGGQGGAGQGGAATAAAAAGGQGGQGGYGGLG
SEGAGQGGYGAGQGGAGAAAAAAAAGGAGGAGRGGLGAGGAGQGYGSGLGGQGGAGQGGAAAAAA
AAGGQGGQGGYGGLGSQGAGQGGAGRGAAAAAAAAGGQGGQGGYGGLGSQGAVQGGAGRGAAAA
AAAAGGQGGQGGYGGLGSQGAGQGGAGRGAAAAAAAAGGQGGQGGYGGLGSEGAGQGGAGRGAAA
AAAAAGGQGGQGGYGGLGSQGAGQGGYGAGQGGARAAAAAAAAGGAGGAGRGGLGAGGAGQGYGS
GLGGQGGAGQGGAAAAAAAAAAGGQGGQGGYGGLGSQGAGQGGYGAGQGGAGAAAAAATAGGAG
GAGRGGLGAGGAGQGYGSGLGGQGGAGQGGAAAAAAAAGGQGGQGGFGRFSSQEAGQGAYGGAYS
GQQGAAASVSAASAAASRLSSPGAASRVSSAVTSLVSSGGPTNPAALSNTISNVVSQISESNPGLSGCDVLVQ
ALLELVSALVHILGSANIGQVNSSAAGQSASLVRQSVYQALS

>Aaur_Masp1b
MIWTVRFSLSLLIVICSQSIFALGQSPWQSASMAESFMTYFSAALGQSGAFTNEQMDDIDTIASSIKMGVDK
MERSGKTSQNKLQAMNMAFASAVADIAIAEGGGQSAQIKTNAIADALASAFLQTTGTVNNQFINEIRGLIS
MFAQANSISSSSASASESTAAAAGGPGGAGQGYGSGLGGAGGVGAASAAAAAGGLGGRGGFGGLGSQG
VGGAGQGAGAAAAAAAAGGDGGAGLRGLGAGQGYGSGLGGAGGAGAASAAAAAGGLGGRGGFGGLG
SQGASGAGQGGAGAAAAAAAAGGDGGAGLRGLGAGQGYGSGLGGAGGAGAASAAAAAGGLGGRGGF
GGLGSQGVGGAGQGAGAAAAAAAAGGDGGAGLRGLGAGQGYGSGLXGAGAGAASAAAAAGGLGGRG
GFGGLGSQGVGGAGQGGAGAAAAAAAAGGDGGAGLRGLGAGQGYGSGLGGAGGAGAASAAAAAGGV
GGRGGFGGLGSQGASGAGQGGAGAASAAAAAGGLGGRGGFGGLGSQGASGAGQGGAGAAAAAAAAG
GDGGAGLRGLGAGQGYGSGLGGAGGAGAASAAAAAGGLGGRGGFGGLGSQGASGAGQGAGAAAAAA
AAGGDGGAGLRGLGAGQGYGSGLGGAGGAGAASAAAAAGGLGGRGGFGGLGSQGVGGAGQGGAGAA
AAAAAAGGDGGAGLRGLGAGQGYGSGLGGAGGAGAASAAAAAGGLGGRGGFGGLGSQGASGAGQGG
AGAAAAAAAAGGDGGAGLRGLGAGQGYGSGLGGAGGAGAASAAAAAAGGDGGAGLRGLGAGQGYGS
GLGGAGGAGAASAAAAAGGLGGRGGFGGLGSQGVGGAGQGAGAAAAAAAAGGDGGAGLRGLGAGQ
GYGSGLGGAGGAGAASAAAAAGGLGGRGGFGGLGSQGASGAGQGGAGAAAAAAAAGGDGGAGLRGL
GAGQGYGSGLGGAGGAGAASAAAAAGGLGGRGGFGGLGSQGASGAGQGGAGAAAAAAAAGGDGGA
GLRGLGAGQGYGSGLGGAGGAGAASAAAAAGGLGGRGGFGGLGSQGASGAGQGGAGAAAAAAAAGG
DGGAGLRGLGAGQGYGSGLGGAGGAGAASAAAAAGGLGGRGGFGGLGSQGASGAGQGGAGAAAAAA
AAGGDGGAGLRGLGAGQGYGSGLGGAGGAGAASAAAAAGGLGGRGGFGGLGSQGVGGAGQGAGAAA
AAAAAGGDGGAGLRGLGAGQGYGSGLGGAGGAGAASAAAAAGGLGGRGGFGGLGSQGVGGAGQGAG
AAAAAAAAGGDGGAGLRGLGAGQGYGSGLGGAGGAGAASAAAAAGGLGGRGGFGGLGSQGASGAGQ
GGAGAAAAAAAAGGDGGAGLRGLGAGQGYGSGLGGAGGAGAASAAAAAGGLGGRGGFGGLGSQGAS
GAGQGGAGAAAAAAAAGGDGGAGLRGLGAGQGYGSGLGGAGGAGAASAAAAAGGLGGRGGFGGLGS
QGASGAGQGAGAAAAAAAAGGDGGAGLRGLGAGQGYGSGLGGAGGAGAASAAAAAGGLGGRGGFGG
LGSQGASGAGQGGAGAAAAAAAAGGDGGAGLRGLGAGQGYGSGLGGAGAASAAAAAGGLGGRGGFG
GLGSQGASGAGQGGAGAAAAAAAAGGDGGAGLRGLGAGQGYGSGLGGAGGAGAASAAAAAGGLGGR
GGFGGLGSQGASGAGQGGAGAAAAAAAAGGDGGAGLRGLGAGQGYGSGLGGAGGAGAASAAAAAGG
LGGRGGFGGLGSQGVGGAGQGAGAAAAAAAAGGDGGAGLRGLGAGQGYGSGLGGAGGAGAASAAAA
AGGLGGRGGFGGLGSQGASGAGQGGAGAASAAAAAGGVGGRGGFGGLGSEGASGAGQGGAGAAAAA
AAAGGDGGAGLRGLGAGQGYGSGLGGAGGAGAASAAAAAGGLGGRGGFGGLGSQGVGGAGQGGAGA
AAAAAAGGDGGAGLRGLGAGQGYGSGLGGAGGAGAASAAAAAAGGDGGAGLRGLGAGQGYGSGLG
GAGGAGAASAAAAAGGLGGRGGFGGLGSQGVGGAGQGAGAAAAAAAAGGDGGAGLRGLGAGQGYGS
GLGGAGAASAAAAAGGLGGRGGFGGLGSQGASGAGQGGAGAAAAAAAAGGDGGAGLRGLGAGQGYG
SGLGGAGGAGAAAAAAAAGGDGGAGLRGLGAGQGYGSGLGGAGGAGAASAAAAAGGLGGRGGFGGL

GSQGASGAGQGAGAAAAAAAAGGDGGAGLRGLGAGQGYGSGLGGAGGAGAASAAAAAGGLGGRGGF
GGLGSQGASGAGQGGAGAAAAAAAAGGDGGAGLRGLGAGQGYGSGLGGAGGAGAASAAAAAGGLGG
RGGFGGLGSQGASGAGQGGAGAAAAAAAAGGDGGAGLRGLGAGQGYGSGLGGAGGAGAASAAAAAG
GLGGRGGFGGLGSQGASGAGQGGAGAAAAAAAAGGDGGAGLRGLGAGQGYGSGLGGAGGAGAASAA
AAAGGLGGRGGFGGLGSQGVGGAGQGAGAAAAAAAGGDGGAGLRGLGAGQGYGSGLGGAGAASAA
AAAGGLGGRGGFGGLGSQGVGGAGQGGAGAAAAAAAAGGDGGAGLRGLGAGQGYGSGLGGAGGAG
AAGAAAAAGGLGGRGGFGGLGSQGVGSAGQGAGAAAAAAAAGGDGGTGLRGLGAGKGYGAGLGGAG
GAGAASAAAAAAGGDGGAGLRGLGAGQGYGSGLGGAGGAGAASAAAAAGGLGGRGGFGGLGSQGVG
GAGQGAGAAAAAAAAGGDGSAGLRGLDAIQGYGSGLGGAGAASAAAAAGGLGGRGGFGGLGSQGASG
AGQGGAGAAAAAAAAGGDGGAGLRGLGAGQGYGSGLGGAGGAGAASAAAAAAGGDGGTGLRGLGAG
QGYGSGLGGAGGAGAASVAAAAGGVGGRGGFGGLGSQGASGAGQGGAGAAAAAAAAGGDGGAGLRG
LGAGKGYGSGLGGAGGAGAASAAAAAAGGDGSAGLRGLDAIQGYGSGLGGAGAASAAAAAGGLGGRGG
FGGLGSQGASGAGQGGAAAAAAAGGDSGAGLRGLGAGQGYGSGLGGAGGAGAASAAAAAGGLGGRG
GFGGLGSQGVGGAGKGAGAAAAAAAAGGDGGAGLRGLGAGQGYGSGLGXAGGAGAASAAAAAGGLG
GRGGFGGLGSQGVGGAGQGAGAAAAAAAAGGDGGAGLRGLGAGQGYGSGLGGAGGAGAASAAAAAG
GLGGRGGFGGLGSQGVGGAGQGAGAAAAAAAAGGDGGAGLRGLGAGQGYGSGLGGAGGAGAASAAA
AAAGGDGSAGLRGLGAGQGYGSGLGGAGGAGAASAAAAAGGLGGRGGFGGLGSQGVGSAGQGAGAA
AAAAAAGGDGGAGLRGLGAGQGYGSGLLGAGAASAAAAAGGLGGRGGFGGLGSQGASGAGQGGAGA
AAAAAAAGGDGGAGLRGLGAGQGYGSGLGGAGGAGAASAAAAAGGLEGRGGFGGLGSQGVGGAVQG
GAGASASAAASGGYGGLGSELEVQGAYRSGAYSGQQSAAVSVAAVSAAASRLSSPNAASRISSAVTSLISGG
GPTNLAALSNTFSNVVYQISVSNPGLSGCDVLVQALLELVSALVHILGSAIIGHVNSSAAGETAALVGQSVYQA
FS

>Aaur_MaSp2.1a
MSCPRLVLAFLALLSTNALFAAAAAATPWDSPALADSFMKSFMDGIGTSGAFTSSQIDDMSTIGDTMMDSV
NRLASSGRISKSKLQALNMAFASSMAEIAATEEGGLSIGAKTSAIASALRGAFLQTTGYANEQFINEITSLINMI
AQANVNAVSASASASAGGGYGAPAYGPSSYGPSQQQSSASSVSVSASAAGAGPRSQAPSRPAQQGPRGY
GPSGPGGTAAASASAGGPGSQGPYGPGQQGPGPRGPSRPSQQGPGGYGPSGPGGASAAAAAAAAGGP
GGQGPYGPGQQGPGAGPYGPGQQGPGQQGPGGYGPSGPGGAAAAAAAAAAGGPGGQGPSGPGQQG
PGGYGPSGPSGASAAAAAAGGQGPYGQGQQGPRGYGPSGPGGTAAAAAAAGGPGGQGQYGPGQQGP
GGYGSSGTGGASAAAAAAAAGGPGGQGPYGPGQQGPYGPGQQGPGGQGRGGYGPSGPGGASAAAAA
AAAGGPGGQGQYGPGQQGPGGYGPSGPGGASAAAAASAAGGPGGQGPSGPGGQGPSGPGQQGPGGY
GPSGPSGASAAAAVAGGQGPYGQGQQGPGGYGPSGPAGASAASAAAAAGGQGGQGPYGPGQQGPYG
PGQQGPGGQGRGGYGPSGPGGASAAAAAAAAGGPGGRGEYGPGQQGPGGYGPSGPGGASAAAAASA
AGGPGGQGPSGPGQQGPGGYGPSGPSGASAAAAVAGGQGPYGQGQQGPGGYGPSGPAGASAASAAAA
AGGQGGQGPYGPGQQRPYGPGQQGPGGQGRGGYGPSGPGGASAAAAAAAAGGPGGQGQYGPGQQG
PGGYGPSGPGGASAAAAASAAGGPGGQGPSGPGQQGPGGYGPSGPSGASAAAAVAGGQGPYGQGQQ
GPGGYGPSGPAGASAASAAAAAGGQGGQGPYTGQQGPYGPGQQGPGGQGRGGYGPSGPGDASAAA
AAAAAGGPGGQGQYGPGQQGPGGYGPSGPGGASAAAAASAAGGPGGQGPSGPGQQGPGGYGPSGPS
GASAAAAVAGGQGPYGQGQQGPGGYGPSGPAGASAASAAAAAGGQGGQGPYGPGQQGPYGPGQQG
PGGQGPGGYGPSGPGGASAAAAAAAAGGPGGQGQYGPGQQGPGGYGPSGPGGASAAAAASAAGGPG
GQGPSGPGQQGPGGYGPSGPSGASAAAAVAVGQGPYGQGQQGPGGYGPSGPAGASAASAAAAAGGQ
GGQGPYGPGQQGPYGPGQQGPGQQGPGGYGPSGPGGSAAAAAAAAAGGPGGQGPSGPGQQGPGGY
GPSGPSGASAAAAAAGGQGPYGQGQQGPGGYGPSGPAGASAASAAAAAGGQGGQGPYGPGQQGPYG

PGQQGPGGQGPGGYGPSGPGGASAAAAAAAAGGSGGQGPYGPGQQGPYGPGQQGPGQQGPGGYGP
SGPSGASAAAAAAGGQGPYGQGQQGPGGYGPSGPAGASAASAAAAAGGQGGQGPYGPGQQGPYGPG
QQGPGGQGPGGYGPSGPGGASAAAAAAAAGGSGGQGPYGPGQQGPYGPGQQGPGQQGPGGYGPSG
PSGASAAAAAAGGQGPYGQGQQGPGGYGPSGPAGASAASAAAAAGGQGGQGPYGPGQQGPYGPGQQ
GPGGQGPGGYGPSGPGGASAAAAAAAAGGSGGQGPYGPGQQGPYGPGQQGPGQQGPGGYGPSGPGG
TAAAAAAAAAGGPAGQGPSGPGQQGPGGYGPSGPSGASAAAAAAGGQGPYGQGQQGPGGYGPSGPA
GASAASAAAAAGGQGGQGPYGPGQQGPYGPGQQGPGGQGPGGYGPSGPGGASAAAAAAAAGGSGGQ
GPYGPGQQGPYGPGQQGPGQQGPGGYGPSGPSGASAAAAVAGGQGPYGQGQQGPGGYGPSGPAGAS
AASAAAAAGGQGGQGPYGPGQQGPYGPGQQGPGGQGPGGYGPSGPGGASAAAAAAAAGGSGGQGPY
GPGQQGPGQQGPGGYGPSGPSGASAAAAAAGGQGPYGQGQQGPGGYGSSGSGGAAAAAATAGGPGG
QGQYGPGQQGPGGYGPSGPGAASAAAAAAAAGGPGGQGLSGPGQQGPGGYGPSGPSGASAAAAVAG
GQGPYGQGQQGPGGYGPSGPAGVSAASAAAAAGGQGGQGPYGPGQQGPYGPGQQGPGGQGPGGYG
PSGPGGASAAAAAAAAGGSGGQGPYGPGQQGPYGPGQQGPGGQGPGGYGPSGPGGSAAAAAAAASAA
AGGAGGQGPSGPGQQGPESYGPSGPSGASAATAAAGGQGPYGQGQQGPGGYGPSGPVSGVSVSVSSAA
SRLSSPAASSRVSSAVSTLASSGPSDAGVVSSALSNLVSQVSTNHPGLSECDVIVQALLELVSALVHILGSSSVG
QVDYNGASYSAQNLGQAVAQALA

>Aaur_MaSp2.1b
MSCPRLVLAFLALLSTHALFAAAAGATPWDSPALADSFMKCFMDGIGTSGAFTSSQIDDMSTIGDTMIDSV
NRLASSGRISKSKLQALNMAFASSMAEIAATEEGGLSIGAKTSAIASALRGAFLQTTGYSNEQFINEITSLVSMI
AQANTNSVSASASASAGGGYGGSSYGPSSVSSVSASASSAGAGPAQQGPGSYGPSGPGGYGPSGSSAAAA
ASGGQGPGNYGSSGSGGAGPSGPGGYGPGSQGSSGPGNQGPGGVSAAAAAASGPGGYGPGSQGSSGPG
GYGPLSQGQSGPGGAGGYGPGGASAAAAAVAASGPAGYGQGSQGPSGTGASGPGGAGGYGPGSQGPG
GAAAAAAASGPGGYGPGSQGPSGPGGFGPGSQGQSGPGGYGPGNQGQSGPSGAGGYGPGGASAAAAA
AAASGPGGYGPGSQGPYGPGSQGPSGPGGYGSGSSGPGGAGGYGPGSQGPGGQGTAAAAAAASGPGG
YGSGNQGPSGTGASGPGGAGGYGPGSQGPGAAAAAAAASGPAGYGPGSQGQSGPGSQGPGGASAAAA
AAASGPGGYGPGSQGPSGPSGYGPGASGPGGAAGYGPGSQGPGAASAAAAAAAASGSGGYGPGSQGPY
GPGSQGPSGSGSQGPSGSGGYGPGASGPGGAGSYGPGSQGPGGASAAAAAAASAPGGYGPGSQGSSGP
SGYGPGASGPGGAGGYGPGSQGPGGASAAAAAAAASGPGGYGPGSQGPYGPGNQGPSGPGSQGPSGS
GGYGPGASGPGGTGSYGPGSQGPGGASAAAAAAASAPGGYGPGSQGLSGPSGYGPGASGPSAGGYGP
GSQGPGGASAAAAAAAASGPGGYGPGSQGPYGPGSQGPSGPGSQGPSGSGGYGPGASGPGGYGPGSQ
GPGGASAAAAAAASGPGGYGPGSQGQSGPGGYGSGSSGPGGAGGYGPGSQGPGGASAAAAAAASGPG
GYGPGSQGPSGPGSQGPSGPGSQGPSGSYGYGPGASGPGGAGSYGPGSQGPGGASAAAAAAASGPGGY
GPGSQGPSGPGSQGPSGSGGYGPGASGPGGYGPGSQGPGGASAAAAAAASGPGGYGPGSQGQSGPGG
YGSGASGPGGAGGYGPGSQGPGGASAAAAAAASGPGGYGPGSQGPSGPGSQGPSGPGSQGPSGSGGYG
PGASGPGGAGSYGPGSQGPGGASAAAAAAASGPEGYGPGSGGPSGPSGYGPGASGPGGTGGYGPGSQG
PGGASAAAAAAASGPGGYGPGSQGPSGPGSQGPSGPGSQGPSGSGGYGPGASGPGGYGPGSQGPGGAS
AAAAAAASGPGGYGPGSQGQSGPGGYGSGSSGPGGAGGYGPGSQGPGGASAAAAAAASGPGGYGPGS
QGPSGPGSQGPSGPGSQGPSGSYGYGPGASGPGGAGSYGPGSQGPGGASAAAAAAASGPGGYGPGSQG
PSGPGSQGPSGSGGYGPGVYGPGSQGPGGASAAAAAAASGPGGYGPGSQGQSGPGGYGSGASGPGGAG
GYGPGSQGPGGASAAAAAAASGPGGYGPGSQGPSGPGSQGPSGPGSQGPSGSGGYGPGASGPGGAGSY
GPGSQGPGGASAAAAAAASGPGGYGPGSGGPSGPSGYGPGASGLGSAGGYGRGSQGPGGASAAAAAAA
SGPGGYGPGSQGPSGPGSQGPSGSGGYGPGASGPGGYGPGSQGPGGASAAAAAAASGPGGYGPGSQG
QSGPGGYGSGSSGPGGAGGYGPGSQGPGGASAAAAAAASGPGGYGPGSQGPSGPGSQGPSGPGSQGPS

GSYGYGPGASGPGGAGSYGPGSQGPGGASAAAAAAASGPGGYGPGSQGPSGPGSQGPSGSGGYGPGAS
GPGVYGPGSQGPGGASAAAAAAASGPGGYGPGSQGQSGPGGYGSGASGPGGAGGYGPGSQGPGGASA
AAAAAASGPGGYGPGSQGSSGPGSQGPSGPGSQGPSGSGGYGPGASGPGGAGSYGPGSQGPGGASAAA
AAAASGPGGYGPGSGGPSGPSGYGPGASGPGSAGGYGPGSQGPGGASAAAAAAASGPGGYGPGSQGPA
GPGSQGPSGPGSQGPSGSGGYGPGASGPGGYGPGSQGPGGASAAAAAAASGPGGYGPGSQGQSGPGG
YGSGSSGPGGAGGYGPGSQGPGGASAAAAAAASGPGGYGPGSQGPSGPGSQGPSGPGSQGPSGSGGYG
PGASGPGGAGSYGPGSQGPGGASAAAAAAASGPGGYGPGSQGPSGPGSQGPSGSGGYGPGASGPGVYG
PGSQGPGGASAAAAAAASGPGGYGPGSQGPSGPGSQGPSGPGSQGPSGSGGYGPGASGPGGAGSYGPG
SQGPGGASAAAAAAASGPGGYGPGSGGPSGPSGYGPGASGPGSAGGYGPGSQGPGGASAAAAAAASGP
GGYGPGSQGPSGPGSQGPSGPGSQGPSGSGGYGPGASGPGGYGPGSQGPGGASAAAAAAASGPGGYGP
GSQGQSGPGGYGSGSSGPGGAGGYGPGSQGPGGASAAAAAAASGPGGYGPGSQGPSGPGSQGPSGPG
GAGAAAAAAAASGPGGYGPGSQGSSGPGSQGPSGSGGYGPGASGSGGYGPGSQGPGGASAAAAAAASG
PGGYGPGSQGPSGPGSQGPSGPSVYGPGASGPGGAGGYGPGSQGPGGASAAAAAASGPGGYGPGSQGP
SGPGSQGPSGPGSQGPSGSGGYGPGASGPGGYGPGSQGPGGASAAAAAAASGPGGYGPGSQGPSGPGS
QGPSGPSGYGPGASGPGGAGGYGPGSQGPGGASAAAAAASGPGGYGPGGQGPSGPGSQGPSGPGGSG
AAAAAAASGPGGYGPGSQGLSGPGSQGSSGLGGYGPGGAGGYGPGSQGPGGASAAAAAAAASGPGGYG
PGSQGPSGPGSQGPSGPGSQGPSGSGGYGPGASGSGGYGPGSQGPGGASAAAAAAASGPGGYGPGSQG
PSGPGSQGPSGPSGYGPGASGPGGAGGYGPGSQGPGGASAAAAAASGPGGYGPGSQGTSGPGSQGPSG
PGSQGPSGSGGYGPGASGPGGYGPGSQGSGGASAAAAAAASGPGGYGPGSQGSSGLGGYGPGGAGGYG
PGSQGPGGASAAAAAAATSGPGGYGPGSQGPSGPGSQGPSGPGSFGPGGAGGYGPSASATVSVAASRLSS
PAASSRVSSTVSSLVSSGPSNGAAVSGALNGLVSQISSSNPGLSGCDVLVQALLELVSALVAILGSANIGSVDYY
SVGQTTQTISQYFS

>Aaur_MaSp2.2a
MNWSIRLALLGFVVLSTQTIFAAGQAATPWENTQLAEDFIISFLRFIGQSGAFSPDQLDDMSTIGETLKTAIEK
MAQSRKSSKSKLQALNMAFASSMAEIAVAEKGGLSLEAKTNAIANALASAFLETTGFVNQQFVSEIKSLIYMI
AQASANEISGSAAAAGGGSGGFGSGQGGYGQGAYASASAASAYGSAPQGAGGPAPQGLSQQGPVRQGP
YGPSAAVAATAVGGRPQGRSASSQQGPSQQGPYGPGAAGAAAAAGGYGPGVGQQGPGDAGQQGPYG
PGAAAVGGYGPGARAGGPQRPIGAGPSLPSARGPQGPGGSGPGSQGPFEPAAAAAAAAAARGFGPGASG
QKGPGEAGQQGPGGAGQQGPGGQGLFGPGAAAAAAAAAGGFGPGAGGQRGPGQQGPGGQGPSGPG
AAAAAAAAAGGFGPGGAGAGPQAGQRGPGGAGAGAAAAAAAGAGGFGPGAGGQQGPGGAGPYGPS
AGGQRGPGGVGQQGPGGQGPFGPGAAAAAAAAAGGFGPGGAGVGPQAAPGQQGPGGAGPYGPGAA
AAAAAAGGFGPGAGGQRGPGQQGLFGPGAAAAAAAAAGGFGPGAGGQKVPGGAGQQGPGGQGPYGP
GAAAAAAAAGGFGPGAGGQRGPGQQGPGGQGPSGPGAAAAAAAAAAGGFGPGGAGAGPQAGQRGP
GGAGAGAAAAAAAGAGGFGPGAGGQQGPGGAGPYGPSAGGQRGPGGVGQQGPGGQGPFGPGAAAA
AAAAAGGFGPGGAGVGPQAAPGQQGPGGAGPYGPGAAAAAAAAAAGGFGPGAGGQRGPGQQGLFGP
GAAAAAAAAAGGFGPGAGGQKVPGGAGQQGPGGQGPYGPGAAAAAAAAGGFGPGAGGQRGPGQQG
PGGQGPSGPGAAAAAAAAAAGGFGPGGAGAGPQAGQRGPGGAGAGAAAAAAAGAGGFGPGAGGQQ
GPGGAGPYGPSAGGQRGPGGVGQQGPGGQGPFGPGAAAAAAAAAGGFGPGGAGVGPQAAPGQQGP
GGAGPYGPGAAAAAAAAAAGGFGPGAGGQRGPGQQGLFGPGAAAAAAAAAGGFGPGAGGQKVPGGA
GQQGPGGQGPYGPGAAAAAAAAGGFGPGAGGQRGPGQQGPGGQGPSGPGAAAAAAAAAAGGFGPG
GAGAGPQAGQRGPGGAGAGAAAAAAAGAGGFGPGAGGQQGPGGAGPYGPSAGGQRGPGGVGQQG
PGGQGPFGPGAAAAAAAAAGGFGPGGAGVGPQAAPGQQGPGGAGPYGPGAAAAAAAAAAGGFGPGA
GGQRGPGQQGLFGPGAAAAAAAAAGGFGPGAGGXKVPGGAGQQGPGGQGPYGPGAAAAAAAAGGFG

PGAGGQRGPGQQGPGGQGPSGPGAAAAAAAAAAGGFGPGGAGAGPQAGQRGPGGAGAGAAAAAAA
GAGGFGPGAGGQQGPGGAGPYGPSAGGQRGPGGVGQQGPGGQGPFGPGAAAAAAAAAGGFGPGGA
GVGPQAAPGQQGPGGAGPYGPGAAAAAAAAGGFGPGAGGQRGPGQQGLFGPGAAAAAAAAAGGFGP
GAGGXKVPGGAGQQGPGGQGPYGPGAAAAAAAAGGFGPGAGGQRGPGQQGPGGQGPSGPGAAAAA
AAAAAGGFGPGGAGAGPQAGQRGPGGAGAGAAAAAAAGAGGFGPGAGGQQGPGGAGPYGPSAGGQ
RGPGGVGQQGPGGQGPFGPGAAAAAAAAAGGFGPGGAGVGPQAAPGQQGPGGAGPYGPGAAAAAA
AAAAGGFGPGAGGQRGPGQQGLFGPGAAAAAAAAAGGFGPGAGGXKVPGGAGQQGPGGQGPYGPGA
AAAAAAAGGFGPGAGGQRGPGQQGPGGQGPSGPGAAAAAAAAAAGGFGPGGAGAGPQAGQRGPGG
AGAGAAAAAAAGAGGFGPGAGGQQGPGGAGPYGPSAGGQRGPGGVGQQGPGGQGPFGPGAAAAAA
AAAGGFGPGGAGVGPQAAPGQQGPGGAGPYGPGAAAAAAAAAAGGFGPGAGGQRGPGQQGLFGPGA
AAAAAAAAGGFGPGAGGQKVPGGAGQQGPGGQGPYGPGAAAAAAAAGGFGPGAGGQRGPGQQGPG
GQGPSGPGAAAAAAAAAAGGFGPGGAGAGPQAGQRGPGGAGAGAAAAAAAGAGGFGPGAGGQQGP
GGAGPYGPSAGGQRGPGGVGQQGPGGQGPFGPGAAAAAAAAAGGFGPGGAGVGPQAAPGQQGPGG
AGPYGPGAAAAAAAAAAGGFGPGAGGQRGPGQQGLFGPGAAAAAAAAAGGFGPGAGGQKVPGGAGQ
QGPGGQGPYGPGAAAAAAAAGGFGPGAGGQRGPGQQGPGGQGPSGPGAAAAAAAAAAGGFGPGGA
GAGPQAGQRGPGGAGAGAAAAAAAGAGGFGPGAGGQQGPGGAGPYGPSAGGQRGPGGVGQQGPG
GQGPFGPGAAAAAAAAAGGFGPGGAGVGPQAAPGQQGPGGAGPYGPGAAAAAAAAAAGGFGPGAGG
QRGPGQQGLFGPGAAAAAAAAAGGFGPGAGGQKVPGGAGQQGPGGQGPYGPGAAAAAAAAGGFGPG
AGGQRGPGQQGPGGQGPSGPGAAAAAAAAAAGGFGPGGAGAGPQAGQRGPGGAGAGAAAAAAAGA
GGFGPGAGGQQGPGGAGPYGPSAGGQRGPGGVGQQGPGGQGPFGPGAAAAAAAAAGGFGPGGAGV
GPQAAPGQQGPGGAGPYGPGAAAAAAAAAAGGFGPGAGGQRGPGQQGLFGPGAAAAAAAAAGGFGP
GAGGQKVPGGAGQQGPGGQGPYGPGAAAAAAAAGGFGPGAGGQRGPGQQGPGGQGPSGPGAAAAA
AAAAAGGFGPGGAGAGPQAGQRGPGGAGAGAAAAAAAGAGGFGPGAGGQQGPGGAGPYGPSAGGQ
RGPGGVGQQGPGGQGPFGPGAAAAAAAAAGGFGPGGAGVGPQAAPGQQGPGGAGPYGPGAAAAAA
AAAAGGFGPGAGGQRGPGQQGLFGPGAAAAAAAAAGGFGPGAGGQKVPGGAGQQGPGGQGPYGPG
AAAAAAAAGGFGPGAGGQRGPGQQGPGGQGPSGPGAAAAAAAAAAGGFGPGGAGAGPQAGQRGPG
GAGAGAAAAAAAGAGGFGPGAGGQQGPGGAGPYGPSAGGQRGPGGVGQQGPGGQGPFGPGAAAAA
AAAAGGFGPGGAGVGPQAAPGQQGPGGAGPYGPGAAAAAAAAAAGGFGPGAGGQRGPGQQGLFGPG
AAAAAAAAAGGFGPGAGGQKVPGGAGQQGPGGQGPYGPGAAAAAAAAGGFGPGAGGQRGPGQQGP
GGQGPSGPGAAAAAAAAAAGGFGPGGAGAGPQAGQRGPGGAGAGAAAAAAAGAGGFGPGAGGQQG
PGGAGPYGPSAGGQRGPGGVGQQGPGGQGPFGPGAAAAAAAAAGGFGPGGAGVGPQAAPGQQGPG
GAGPYGPGAAAAAAAAAAGGFGPGAGGQRGPGQQGLFGPGAAAAAAAAAGGFGPGAGGQKVPGGAG
QQGPGGQGPYGPGAAAAAAAAGGFGPGAGGQRGPGQQGPGGQGPSGPGAAAAAAAAAAGGFGPGG
AGAGPQAGQRGPGGAGAGAAAAAAAGAGGFGPGAGGQQGPGGAGPYGPSAGGQRGPGGVGQQGP
GGQGPFGPGAAAAAAAAAGGFGPGGAGVGPQAAPGQQGPGGAGPYGPGAAAAAAAAGGFGPGAGGQ
RGPGQQGPGGQGLFGPGAAAAAAAAAGGFGPGAGGQKGPGGAGQQGPGGQGPYGPGAAAAAAAAG
GFGPGTGGQRGPGQQVPGGQGPSGPGAAAAAAAAAGGFGPGGAGPGPKAGQGGARFYRPGAAVATAA
VGGYGPGAGQQGPAAPSQQGPGRQIPYGPGAAAAVVGVYAPVPQRPTASAAASRLASPEASSRVSSAVSS
LVSSGPTNPAALSNTISSVVSQISASNPGLSGCDVLVQALLEIVSALVHILGYSSIGQINYGAASQYARLVGQSV
AQALG

>Aaur_MaSp2.2b
MNWSIRLALLGFVVLSTQTVFSAGQGATPWENSQLAEDFINSFLRFIAQSGAFSPNQLDDMSSIGDTLKTAIE
KMAQSRKSSKSKLQALNMAFASSMAEIAVAEQGGLSLEAKTNAIANALTSAFLETTGVVNQQFVSEIKSLIYM

IAQASSNEISGSAAAAGGGSGGGGGSGQGGYGQGAYASASAAAAYGSAPQGAGGPAPQGPSQQGPVSQ
GPYGPGAAAAAAAAGGYGPGAGQQRQQGPGRQGKAGAGQQGPGGQGPYGPSAAAAAAAAGGYGQG
AGQQGPGGAGQQGPGSQRPGGAGQQGPGGQGPYGPAAAAAAAAVGGYGPGAGQQGPGSQGPGSG
GQQGPGSGGQQGPGGQGPYGSGQQGPGGAGQQGPGGQGPYGPGAAAAAAAAGGYGPGAGQQGPG
GAGQQGPGSQGPGGAGQRGPGGQGPYGPGAAAAAAAAGGYGPGAGQQGPGSQGPGSGGQQGPGG
QGPYGPSAAAAAAAAGGYGPGAGQQGRGSGGQQGPGSGGQQGPGGQGPYGSGQQGPGGAGQQGP
GGQGPYGPGAAAAAAAAGGYGPGAGQQGPGGAGQQGPGSQGPGGAGQRGPGGQGPYGPGAAAAAA
AAAGYGPGAGQQGPGSQGPGSGGQQGPGGQGPYGPSAAAAAAAAGGYGPGAGQQGRGSGGQQGPG
SGGQQGPGGQGPYGSGQQGPGGAGQQGPGGQGPYGPGAAAAAAAAGGYGPGAGQQGPGGAGQQG
PGGAGQQGPGSQGPGGAGQRGPGGQGPYGPGAAAAAAAAAGYGPGAGQQGPGSQGPGSGGQQGPG
GQGPYGPSAAAAAAAAGGYGPGAGQQGRGSGGQQGPGSGGQQGPGGQGPYGSGQQGPGGAGQQG
PGGQGPYGPGAAAAAAAAGGYGPGAGQQGPGGAGQQGPGGAGQQGPGGAGQQGPGSQGPGGAGQ
RGPGGQGPYGPGAAAAAAAAGGYGPGAGQQGPGSQGPGSGGQQGPGGQGPYGPSAAAAAAAAGGYG
PGAGQQGRGSGGQQGPGSGGQQGPGGQGPYGSGQQGPGGAGQQGPGGQGPYGPGAAAAAAAAGG
YGPGAGQQGPGGAGQQGPGSQGPGGAGQRGPGGQGPYGPGAAAAAAAAGGYGPGAGQQGPGGAG
QQGPGGAGQQGPGSQGPGGAGQRGPGGQGPYGPGAAAAAAAAGGYGPGAGQQGPGSQGPGSGGQ
QGPGGQGPYGPSAAAAAAAAGGYGPGAGQQGPGGAGQQGPGSQGPGGAGQRGPGGQGPYGPGAAA
AAAAAGGYGPGAGQQGPGSQGPGSGGQQGPGGQGPYGPSAAAAAAAAGGYGPGAGQQGPGSQGPG
SGGQQGPGGQGPYGPSAAAAAAAAGGYGPGAGQQGPGSGGQQGPGGQGPYGSGQQGPGGAGQQGP
GGQGPYGPGAAAAAAAAGGYGPGAGQQGPGGAGQQGPGGAGQQGPGSQGPGGAGQRGPGGQGPY
GPGAAAAAAAAGGYGPGAGQQGPGSQGPGSGGQQGPGGQGPYGPSAAAAAAAAGGYGPGAGQQGP
GGAGQQGPGSQGPGGAGQRGPGGQGPYGPGAAAAAAAAGGYGPGAGQQGPGSQGPGSGGQQGPG
GQGPYGPSAAAAAAAAGGYGPGAGQQGPGSQGPGSGGQQGPGGQGPYGPSAAAAAAAAGGYGPGAG
QQGPGSGGQQGPGGQGPYGSGQQGPGGAGQQGPGGQGPYGPGAAAAAAAAGGYGPGAGQQGPGG
AGQQGPGSQGPGGAGQRGPGGQGPYGPGAAAAAAAAGGYGPGAGQQGPGSQGPGSGGQQGPGGQ
GTYGPSAAAAAAAAGGYGPGAGQQGPGGAGQQGPGSQGPGGAGQRGPGGQGPYGPGAAAAAAAAG
GYGPGAGQQGPGSQGPGSGGQQGPGGQGPYGPSAAAAAAAAGGYGPGAGQQGPGSGGQQGPGGQG
PYGSGQQGPGGAGQQGPGGQGPYGPGAAAAAAAAGGYGPGAGQQGPGGAGQQGPGSQGPGGAGQ
RGPGGQGPYGPGAAAAAAAAGGYGPGAGQQGPGSQGPGSGGQQGPGGQGPYGPSAAAAAAAAGGYG
PGAGQQGPGGAGQQGPGSQGPGGAGQRGPGGQGPYGPGAAAAAAAAGGYGPGAGQQGPGSQGPGS
GGQQGPGGQGPYGPSAAAAAAAAGGYGPGAGQQGPGSQGPGSGGQQGPGGQGPYGPSAAAAAAAA
GGYGPGAGQQGPGSGGQQGPGGQGPYGSGQQGPGGAGQQGPGGQGPYGPGAAAAAAAAGGYGPG
AGQQGPGGAGQQGPGSQGPGGAGQRGPGGQGPYGPGAAAAAAAAGGYGPGAGQQGPGSQGPGSG
GQQGPGGQGPYGPSAAAAAAAAGGYGPGAGQQGPGGAGQQGPGSQGPGGAGQRGPGGQGPYGPGA
AAAAAAAGGYGPGAGQQGPGSQGPGSGGQQGPGGQGPYGPSAAAAAAAAGGYGPGAGQQGPGSQG
PGSGGQQGPGGQGPYGPSAAAAAAAAGGYGPGAGQQGPGSGGQQGPGGQGPYGSGQQGPGGAGQQ
GPGGQGPYGPGAAAAAAAAGGYGPGAGQQGPGGAGQQGPGSQGPGGAGQRGPGGQGPYGPGAAAA
AAAAGGYGPGAGQQGPGSQGPGSGGQQGPGGQGPYGPSAAAAAAAAGGYGPGAGQQGPGGAGQQG
PGSQGPGGAGQRGPGGQGPYGPGAAAAAAAAGGYGPGAGQQGPGSQGPGSGGQQGPGGQGPYGPS
AAAAAAAAGGYGPGAGQQGPGSQGPGSGGQQGPGGQGPYGPSAAAAAAAAGGYGPGAGQQGPGSG
GQQGPGGQGPYGSGQQGPGGAGQQGPGGQGPYGPGAAAAAAAAGGYGPGAGQQGPGGAGQQGPG
SQGPGGAGQRGPGGQGPYGPGAAAAAAAAGGYGPGAGQQGPGSQGPGSGGQQGPGGQGPYGPSAA
AAAAAAGGYGPGAGQQGPGGAGQQGPGSQGPGGAGQRGPGGQGPYGPGAAAAAAAAGGYGPGAGQ
QGPGSQGPGSGGQQGPGGQGPYGPSAAAAAAAAGGYGPGAGQQGPGSQGPGSGGQQGPGGQGPYG

PSAAAAAAAAGGYGPGAGQQGPGSGGQQGPGGQGPYGSGQQGPGGAGQQGPGGQGPYGPGAAAAA
AAAGGYGPGAGQQGPGGAGQQGPGSQGPGGAGQRGPGGQGPYGPGAAAAAAAAGGYGPGAGQQGP
GSQGPGSGGQQGPGGQGPYGPSAAAAAAAAGGYGPGAGQQGPGGAGQQGPGSQGPGGAGQRGPGG
QGPYGPGAAAAAAAAGGYGPGAGQQGPGSQGPGSGGQQGPGGQGPYGPSAAAAAAAAGGYGPGAG
QQGPGSQGPGSGGQQGPGGQGPYGPSAAAAAAAAGGYGPGAGQQGPGSGGQQGPGGQGPYGSGQQ
GPGGAGQQGPGGQGPYGPGAAAAAAAAGGYGPGAGQQGPGGAGQQGPGSQGPGGAGQRGPGGQG
PYGPGAAAAAAAAGGYGPGAGQQGPGSQGPGSGGQQGPGGQGPYGPSAAAAAAAAGGYGPGAGQQ
GPGGAGQQGPGSQGPGGAGQRGPGGQGPYGPGAAAAAAAAGGYGPGAGQQGPGSQGPGSGGQQGP
GGQGPYGPSAAAAAAAAGGYGPGAGQQGPGSQGPGSGGQQGPGGQGPYGPSAAAAAAAAGGYGPGA
GQQGPGSGGQQGPGGQGPYGSGQQGPGGAGQQGPGGQGPYGPGAAAAAAAAGGYGPGAGQQGPG
GAGQQGPGGAGQQGPGSQGPGGAGQQGPGGQGPYGPGAAAAAAAAGGYGPGAGQQGPGSQGPGS
GGQQGPGGQGPYGPSAAAAAAAAGGYGPGAGQQGPGGAGQQGLGSQGPGGAGQRGPGGQGPYGPG
AAAAAAAAGGYGPGAGQQGPGSQGPVASAAASRLSSPQASSRVSSAVSTLVSSGPTNPAALSNAISNVVSQ
VSASNPGLSGCDVLVQALLEIVSALVHILGSSSIGQINYAASSQYAQMVGNSVTQALG

>Aaur_MaSp2.2c
MNWSIRLALLGFVVLSTQTVFXAGQGATPWENSQLAEDFINSFLRFIAQSGAFSPNQLDDMSSIGDTLKTAIE
KMAQSRKSSKSKLQALNMAFASSMAEIAVAEQGGLSLEAKTNAIANALTSAFLETTGVVNQQFVSEIKSLIYM
IAQASSNEISGSAAAAGGGSGGGGGSGQGGYGQGAYASASAAAAYGSAPQGAGGPAPQGPSQQGPVSQ
GPYGPGAAAAAAAAGGYGPGAGQQRQQGPGRQGKAGAGQQGPGGQGPYGPSAAAAAAAAGGYGPG
AGQQGPGSQGPGAGQQGPGSQGPGSGGQQGPGGQGPYGPSAAAAAAAAGGYGSGAGQQGPGSQGP
GSGGQQGPGGQGPYGPSAAAAAAAAGGYGPGAGQQGPGSGGQQGPGGQGPYGSGQQGPGGAGQQ
GPGGQGPYGPGAAAAAAAAGGYGPGAGQQGPGGAGQQGPGSQGPGGAGQRGPGGQGPYGPGAAAA
AAAAGGYGPGAGQQGPGSQGPGSGGQQGPGGQGPYGPSAAAAAAAAGGYGPGAGQQGPGSGGQQG
PGGQGPYGSGQQGPGGAGQQGPGGQGPYGPGAAAAAAAAGGYGPGAGQQGPGGAGQQGPGSQGP
GGAGQRGPGGQGPYGPGAAAAAAAAGGYGPGAGQQGPGSQGPGSGGQQGPGGQGPYGPSAAAAAA
AAGGYGPGAGQQGPGSQGPGSGGQQGPGGQGPYGPSAAAAAAAAGGYGPGAGQQGPGSGGQQGPG
GQGPYGSGQQGPGGAGQQGPGGQGPYGPGAAAAAAAAGGYGPGAGQQGPGGAGQQGPGSQGPGG
AGQRGPGGQGPYGPGAAAAAAAAGGYGPGAGQQGPGSGGQQGSGGQGPYGPSAAAAAAAAGGYGPR
AGQQGPGSQGPGGAGQQGPGGQGPYGPGAAAAAAAAGGYGPGAGQQGPGSQGPGSGGQQGPGGQ
GPYGPSAAAAAAAAGGYGPGAGQQGPGSQGPGSGGQQGPGGQGPYGPSAAAAAAAAGGYGPGAGQQ
GPGSGGQQGPGGQGPYGSGQQGPGGAGQQGPGGQGPYGPGAAAAAAAAGGYGPGAGQQGPGGAG
QQGPGSQGPGGAGQQGPGGQGPYGPGAAAAAAAAGGYGPGAGQQGPGSQGPGSGGQQGPGGQGP
YGPSAAAAAAAAGGYGPGAGQQGPGSQGPGSGGQQGPGGQGPYGPSAAAAAAAAGGYGPGAGQQGP
GSGGQQGPGGQGPYGSGQQGPGGAGQQGPGGQGPYGPGAAAAAAAAGGYGPGAGQQGPGGAGQQ
GPGSQGPGGAGQRGPGGQGPYGPGAAAAAAAAGGYGPGAGQQGPGSGGQQGSGGQGPYGPSAAAA
AAAAGGYGPRAGQQGPGSQGPGGAGQQGPGGQGPYGPGAAAAAAAAGGYGPGAGQQGPGSQGPGS
GGQQGPGGQGPYGPSAAAAAAAAGGYGPGAGQQGPGSQGPGSGGQQGPGGQGPYGPSAAAAAAAA
GGYGPGAGQQGPGSGGQQGPGGQGPYGSGQQGPGGAGQQGPGGQGPYGPGAAAAAAAAGGYGPG
AGQQGPGGAGQQGPGSQGPGGAGQQGPGGQGPYGPGAAAAAAAAGGYGPGAGQQGPGSQGPGSG
GQQGPGGQGPYGPSAAAAAAAAGGYGPGAGQQGPGSQGPGSGGQQGPGGQGPYGPSAAAAAAAAG
GYGPGAGQQGPGSGGQQGPGGQGPYGSGQQGPGGAGQQGPGGQGPYGPGAAAAAAAAGGYGPGA
GQQGPGGAGQQGPGSQGPGGAGQRGPGGQGPYGPGAAAAAAAAGGYGPGAGQQGPGSGGQQGPG
GQGPYGPSAAAAAAAAGGYGPRAGQQGPGSQGPGGAGQQGPGGQGPYGPGAAAAAAAAGGYGPGAG

QQGPGSQGPGSGGQQGPGGQGPYGPSAAAAAAAAGGYGPGAGQQGPGSQGPGSGGQQGPGGQGPY
GPSAAAAAAAAGGYGPGAGQQGPGSGGQQGPGGQGPYGSGQQGPGGAGQQGPGGQGPYGPGAAAA
AAAAGGYGPGAGQQGPGGAGQQGPGSQGPGGAGQRGPGGQGPYGPGAAAAAAAAGGYGPGAGQQ
GPGSGGQQGSGGQGPYGPSAAAAAAAAGGYGPRAGQQGPGSQGPGGAGQQGPGGQGPYGPGAAAA
AAAAGGYGPGAGQQGPGSQGPGSGGQQGPGGQGPYGPSAAAAAAAAGGYGPGAGQQGPGSQGPGS
GGQQGPGGQGPYGPSAAAAAAAAGGYGPGAGQQGPGSGGQQGPGGQGPYGSGQQGPGGAGQQGP
GGQGPYGPGAAAAAAAAGGYGPGAGQQGPGGAGQQGPGSQGPGGAGQQGPGGQGPYGPGAAAAAA
AAGGYGPGAGQQGPGSQGPGSGGQQGPGGQGPYGPSAAAAAAAAGGYGPGAGQQGPGSQGPGSGG
QQGPGGQGPYGPSAAAAAAAAGGYGPGAGQQGPGSGGQQGPGGQGPYGSGQQGPGGAGQQGPGG
QGPYGPGAAAAAAAAGGYGPGAGQQGPGGAGQQGPGSQGPGGAGQRGPGGQGPYGPGAAAAAAAA
GGYGPGAGQQGPGSGGQQGSGGQGPYGPSAAAAAAAAGGYGPRAGQQGPGSQGPGGAGQQGPGGQ
GPYGPGAAAAAAAAGGYGPGAGQQGPGSQGPGSGGQQGPGGQGPYGPSAAAAAAAAGGYGPGAGQ
QGPGSQGPGSGGQQGPGGQGPYGPSAAAAAAAAGGYGPGAGQQGPGSGGQQGPGGQGPYGSGQQG
PGGAGQQGPGGQGPYGPGAAAAAAAAGGYGPGAGQQGPGGAGQQGPGSQGPGGAGQQGPGGQGP
YGPGAAAAAAAAGGYGPGAGQQGPGSQGPGSGGQQGPGGQGPYGPSAAAAAAAAGGYGPGAGQQG
PGSQGPGSGGQQGPGGQGPYGPSAAAAAAAAGGYGPGAGQQGPGSGGQQGPGGQGPYGSGQQGPG
GAGQQGPGGQGPYGPGAAAAAAAAGGYGPGAGQQGPGGAGQQGPGSQGPGGAGQQGPGGQGPYG
PGAAAAAAAAGGYGPGAGQQGPGSQGPGSGGQQGPGGQGPYGPSAAAAAAAAGGYGPGAGQQGPG
SQGPGSGGQQGPGGQGPYGPSAAAAAAAAGGYGPGAGQQGPGSGGQQGPGGQGPYGSGQQGPGGA
GQQGPGGQGPYGPGAAAAAAAAGGYGPGAGQQGPGGAGQQGPGSQGPGGAGQQGPGGQGPYGPG
AAAAAAAAGGYGPGAGQQGPGSQGPGSGGQQGPGGQGPYGPSAAAAAAAAGGYGPGAGQQGPGSQ
GPGSGGQQGPGGQGPYGPSAAAAAAAAGGYGPGAGQQGPGSGGQQGPGGQGPYGSGQQGPGGAGQ
QGPGGQGPYGPGAAAAAAAAGGYGPGAGQQGPGGAGQQGPGSQGPGGAGQQGPGGQGPYGPGAA
AAAAAAGGYGPGAGQQGPGSQGPGSGGQQGPGGQGPYGPSAAAAAAAAGGYGPGVGQQGPGSQGP
GSGGQQGPGGQGPYGPSAAAAAAAAGGYGPGAGQQGPGSGGQQGPGGQGPYGSGQQGPGGAGQQ
GPGGQGPYGPSAAAAAAAAGGYGPGAGQQVLGSQGPVASAAASRLSSPQASSRVSSAVSTLVSSGPTNPA
ALSNAISNVVSQVSASNPGLSGCDVLVQALLEIVSALVHILGSSSIGQINYAASSQYAQMVGNSVTQALG

>Aaur_MaSp2.2d
MNWSIRLALLGFVVLSTQTVFSAGQGATPWENSQLAEEFINSFLRFIAQSGAFSPNQLDDMSSIGDTLKTAIE
KMAQSRKSSKSKLQALNMAFASSMAEIAVAEQGGLSLESKTNAIANALASAFLETTGFVNQQFVSEIKSLIYM
IAQASSNEISGSAAAAGGGSGGGGGSGQGGYGQGAYASASAAAAYGSAPQGAGGPAPQGPSQQGPVSQ
GPYGPGAAVAAAAAGGYGPGAGQQGQQGPGRQGNAGPGQQGPGGQGPYGPSAAAAAAAAGGYGPG
AGQQGPGGAGQQGPGSQGPGGAGQQGPGGQGPYGPGAAAAAAAVGGYGPGAGQQGPGSQGPGSG
GQQGPGGQGPYGPSAAAAAAAAGGYGPGAGQQGPGSQGPGSGGQQGPGGQGPYGPSAAAAAAAAG
GYGPGAGQQGPGSGGQQGPGGQGPYGSGQQGPGGAGQQGPGGQGPYGPGAAAAAAAAGGYGPGA
GQQGPGGAGQQGPGSQGPGGAGQQGPGGQGPYGPGAAAAAAAVGGYGPGAGQQGPGSQGPGRGG
QQGPGGQGPYGPSAAAAAAAAGGYGPGAGQQGPGSQGPGSGGQQGPGGQGPYGPSAAAAAAAAGGY
GPGAGQQGPGSQGPGSGGQQGPGGQGPYGPSAAAAAAAAGGYGPGAGQQGPGSGGQQGPGGQGPY
GSGQQGPGGAGQQGPGGQGPYGPGAAAAAAAAGGYGPGAGQQGPGGAGQQGPGSQGPGGAGQQG
PGGQGPYGPGAAAAAAAVGGYGPGAGQQGPGSQGPGSGGQQGPGGQGPYGPSAAAAAAAAGGYGPG
AGQQGPGSQGPGSGGQQGPGGQGPYGPSAAAAAAAAGGYGPGAGQQGPGSGGQQGPGGQGPYGSG
QQGPGGAGQQGPGGQGPYGPGAAAAAAAAGGYGPGAGQQGPGGAGQQGPGSQGPGGAGQQGPGG
QGPYGPGAAAAAAAVGGYGPGAGQQGPGSQGPGSGGQQGPGGQGPYGPSAAAAAAAAGGYGPGAGQ

QGPGSQGPGSGGQQGPGGQGPYGPSAAAAAAAAGGYGPGAGQQGPGSGGQQGPGGQGPYGSGQQG
PGGAGQQGPGGQGPYGPGAAAAAAAAGGYGPGAGQQGPGGAGQQGPGSQGPGGAGQQGPGGQGP
YGPGAAAAAAAVGGYGPGAGQQGPGSQGPGSGGQQGPGGQGPYGPSAAAAAAAAGGYGPGAGQQG
PGSQGPGSGGQQGPGGQGPYGPSAAAAAAAAGGYGPGAGQQGPGSGGQQGPGGQGPYGSGQQGPG
GAGQQGPGGQGPYGPGAAAAAAAAGGYGPGAGQQGPGGAGQQGPGSQGPGGAGQQGPGGQGPYG
PGAAAAAAAVGGYGPGAGQQGPGSQGPGSGGQQGPGGQGPYGPSAAAAAAAAGGYGPGAGQQGPGS
QGPGSGGQQGPGGQGPYGPSAAAAAAAAGGYGPGAGQQGPGSGGQQGPGGQGPYGSGQQGPGGAG
QQGPGGQGPYGPGAAAAAAAAGGYGPGAGQQGPGGAGQQGPGSQGPGGAGQQGPGGQGPYGPGA
AAAAAAVGGYGPGAGQQGPGSQGPGSGGQQGPGGQGPYGPSAAAAAAAAGGYGPGAGQQGPGSQG
PGSGGQQGPGGQGPYGPSAAAAAAAAGGYGPGAGQQGPGSGGQQGPGGQGPYGSGQQGPGGAGQQ
GPGGQGPYGPGAAAAAAAAGGYGPGAGQQGPGGAGQQGPGSQGPGGAGQQGPGGQGPYGPGAAAA
AAAVGGYGPGAGQQGPGSQGPGSGGQQGPGGQGPYGPSAAAAAAAAGGYGPGAGQQGPGSQGPGS
GGQQGPGGQGPYGPSAAAAAAAAGGYGPGAGQQGPGSGGQQGPGGQGPYGSGQQGPGGAGQQGP
GGQGPYGPGAAAAAAAAGGYGPGAGQQGPGGAGQQGPGSQGPGGAGQQGPGGQGPYGPGAAAAAA
AVGGYGPGAGQQGPGSQGPGSGGQQGPGGQGPYGPSAAAAAAAAGGYGPGAGQQGPGSQGPGSGG
QQGPGGQGPYGPSAAAAAAAAGGYGPGAGQQGPGSQGPGSGGQQGPGGQGPYGPSAAAAAAAAGGY
GPGAGQQGPGSGGQQGPGGQGPYGSGQQGPGGAGQQGPGGQGPYGPGAAAAAAAAGGYGPGAGQ
QGPGGAGQQGPGSQGPGGAGQQGPGGQGPYGPGAAAAAAAVGGYGPGAGQQGPGSQGPGSGGQQ
GPGGQGPYGPSAAAAAAAAGGYGPGAGQQGPGSQGPGSGGQQGPGGQGPYGPSAAAAAAAAGGYGP
GAGQQGPGSQGPGSGGQQGPGGQGPYGPSAAAAAAAAGGYGPGAGQQGPGSGGQQGPGGQGPYGS
GQQGPGGAGQQGPGGQGPYGPGAAAAAAAAGGYGPGAGQQGPGGAGQQGPGSQGPGGAGQQGPG
GQGPYGPGAAAAAAAVGGYGPGAGQQGPGSQGPGSGGQQGPGGQGPYGPSAAAAAAAAGGYGPGAG
QQGPGSQGPGSGGQQGPGGQGPYGPSAAAAAAAAGGYGPGAGQQGPGSGGQQGPGGQGPYGSGQQ
GPGGAGQQGPGGQGPYGPGAAAAAAAAGGYGPGAGQQGPGGAGQQGPGSQGPGGAGQQGPGGQG
PYGPGAAAAAAAVGGYGPGAGQQGPGSQGPGSGGQQGPGGQGPYGPSAAAAAAAAGGYGPGAGQQ
GPGSQGPGSGGQQGPGGQGPYGPSAAAAAAAAGGYGPGAGQQGPGSGGQQGPGGQGPYGSGQQGP
GGAGQQGPGGQGPYGPGAAAAAAAAGGYGPGAGQQGPGGAGQQGPGSQGPGGAGQQGPGGQGPY
GPGAAAAAAAVGGYGPGAGQQGPGSQGPGSGGQQGPGGQGPYGPSAAAAAAAAGGYGPGAGQQGP
GSQGPGSGGQQGPGGQGPYGPSAAAAAAAAGGYGPGAGQQGPGSGGQQGPGGQGPYGSGQQGPGG
AGQQGPGGQGPYGPGAAAAAAAAGGYGPGAGQQGPGGAGQQGPGSQGPGGAGQQGPGGQGPYGP
GAAAAAAAVGGYGPGAGQQGPGSQGPGSGGQQGPGGQGPYGPSAAAAAAAAGGYGPGAGQQGPGS
QGPGSGGQQGPGGQGPYGPSAAAAAAAAGGYGPGAGQQGPGSGGQQGPGGQGPYGSGQQGPGGAG
QQGPGGQGPYGPGAAAAAAAAGGYGPGAGQQGPGGAGQQGPGSQGPGGAGQQGPGGQGPYGPGA
AAAAAAVGGYGPGAGQQGPGSQGPGSGGQQGPGGQGPYGPSAAAAAAAAGGYGPGAGQQGPGSQG
PGSGGQQGPGGQGPYGPSAAAAAAAAGGYGPGAGQQGPGSQGPGSGGQQGPGGQGPYGPSAAAAAA
AAGGYGPGAGQQGPGSGGQQGPGGQGPYGSGQQGPGGAGQQGPGGQGPYGPGAAAAAAAAGGYGP
GAGQQGPGGAGQQGPGSQGPGGAGQQGPGGQGPYGPGAAAAAAAVGGYGPGAGQQGPGSQGPGS
GGQQGPGGQGPYGPSAAAAAAAAGGYGPGAGQQGPGSQGPGSGGQQGPGGQGPYGPSAAAAAAAA
GGYGPGAGQQGPGSQAPVASAAASRLSSPQASSRVSSAVSTLVSSGPTNPAALSNAISSVVSQVSASNPGLS
GCDVLVQALLELVSALVHILGSSSIGQINYAASSQYAQMVGNSVTQALG

>Aaur_MaSp2.2e
MNWSIRLALLGLVVLSTQTVFSAGQGATPWENSQLAEEFINSFLRFIAQSGAFSPNQLDDMSSIGDTLKTAIE
KMAQSRKSSKSKLQALNMAFASSMAEIAVAEQGGLSLEAKTNAIANALTSAFLETTGVVNQQFVSEIKGLIY

MIAQASSNEISGSAAAAGGGSGGGSSGQGGYGQGAYASVSTATTYGSAPQGAGGPAPQGPSQQGPISQP
SYGASATVTVTTVGGRQQGPTGPSQQGPGQQGPYGPSAAAASAAVSGYGPGGGQQEQQGPGGQGPGS
AGQQGPGQQGPYGSSAAATAAAAGGYGPGAGQQGPGRAGQQGPGSQGPGGAGQQGPGGQGPYGPG
ADAAAAAVGGYGPGAGQQGPGSQGPGSGGQQGPGGQGPYGPSAAAAAAATAGYGPGAGQQGPGSQ
GPGGAGQQGPGGQGPYGPGSAAAAAAAGGYGPGVGQQGPGSQGPGSSGQQGPGGQGPYGPSAAAAA
AAAGGYGPGAGQQGPGGAGQQGPGSQGPGGAGQQGPGGQGPYGPGAAASAAAVGGYGPGAGQQG
PGSQGPGSGGQQGPGGQGPYAPSAAAAAAATGGYGPGAGQQGPGSQGPGSGGQQGPGSQGPYGPSA
ATAAAAAGGYGPGAGQQGPGSQGPGSGGQQGPGSQGPYGPSAAAAAAAAGGYGPGAGQQGPGSGGQ
QGSGGQGPYGSGQQGPGGAGQQGPGGQGPYGPGSAAAAAAAGGYGPGVGQQGPGSQGPGSSGQQG
PGGQGPYGPSAAAAAAAAGGYGPGAGQQGPGGAGQQGPGSQGPGGAGQQGPGGQGPYGPGAAAAA
AAVGGYGPGAGQQGPGSQGPGSGGQQGPGGQGPYGPSAAAAAAATGGYGPGAGQQGPGSQGPGSG
GQQGPGGQGAYGSSAAAAAAAAGGYGPGAGQQGPGSGGQQGSGGQGPYGSGQQGPGGAGQQGPG
GQGPYGPGSAAAAAAAGGYGPGVGQQGPGSQGPGSSGQQGPGGQGPYGPSAAAAAAAAGGYGPGAG
QQGPGGAGQQGPGSQGPGGAGQQGPGGQGPYGPGAAAAAAAVGGYGPGAGQQGPGSQGPGSGGQ
QGPGGQGPYGPSAAAAAAATGGYGPGAGQQGPGSQGPGSGGQQGPGGQGPYGSSAAAAAAAAGGYG
PGAGQQGPGSGGQQGSGGQGPYGSGQQGPGGAGQQGPGGQGPYGPGSAAAAAAAGGYGPGVGQQ
GPGSQGPGSSGQQGPGGQGPYGPSAAAAAAAAGGYGPGAGQQGPGGAGQQGPRSQGPGGAGQQGP
GGQGPYGPGAAAAAAAVGGYGPGAGQQGPGSQGPGSGGQQGPGGQGPYGPSAAAAAAATGGYGPGA
GQQGPGSQGPGSGGQQGPGGQGAYGSSAAAAAAAAGGYGPGAGQQGPGSGGQQGSGGQGPYGSGQ
QGPGGAGQQGPGGQGPYGPGSAAAAAAAGGYGPGVGQQGPGSQGPGSSGQQGPGGQGPYGPSAAA
AAAAAGGYGPGAGQQGPGGAGQQGPGSQGPGGAGQQGPGGQGPYGPGAAAAAAAVGGYGPGAGQ
QGPGSQGPGSGGQQGPGGQGPYGPSAAAAAAATGGYGPGAGQQGPGSQGPGSGGQQGPGGQGAYG
SSAAAAAAAAGGYGPGAGQQGPGSGGQQGSGGQGPYGSGQQGPGGAGQQGPGGQGPYGPGSAAAA
AAAGGYGPGVGQQGPGSQGPGSSGQQGPGGQGPYGPSAAAAAAAAGGYGPGAGQQGPGGAGQQGP
GSQGPGGAGQQGPGGQGPYGPGAAAAAAAVGGYGPGAGQQGPGSQGPGSGGQQGPGGQGPYGPSA
AAAAAATGGYGPGAGQQGPGSQGPGSGGQQGPGGQGAYGSSAAAAAAAAGGYGPGAGQQGPGSGG
QQGSGGQGPYGSGQQGPGGAGQQGPGGQGPYGPGSAAAAAAAGGYGPGVGQQGPGSQGPGSSGQQ
GPGGQGPYGPSAAAAAAAAGGYGPGAGQQGPGGAGQQGPGSQGPGGAGQQGPGGQGPYGPGAAAA
AAAVGGYGPGAGQQGPGSQGPGSGGQQGPGGQGPYGPSAAAAAAATGGYGPGAEQQGPGSQGPGSG
GQQGPGGQGAYGSSAAAAAAAAGGYGPGAGQQGPGSGGQQGSGGQGPYGSGQQGPGGAGQQGPG
GQGPYGPGSAAAAAAAGGYGPGVGQQGPGSQGPGSSGQQGPGGQGPYGPSAAAAAAAAGGYGPGAG
QQGPGGAGQQGPGSQGPGGAGQQGPGGQGPYGPGAAAAAAAVGGYGPGAGQQGPGSQGPGSGGQ
QGPGGQGPYGPSAAAAAAATGGYGPGAEQQGPGSQGPGSGGQQGPGGQGAYGSSAAAAAAAAGGYG
PGAGQQGPGGQGPYGSGQQGPGGAGQQGPGGQGPYGPGSAAAAAAAGGYGPGDGQQGPGGAGQQ
GPGSQGPGGAGQQRPGGQGPYGPGAAAAAAAVGGYGPGAGQQGPGSQGPGSGGQLGPGSQGLYGPS
SAAAAAAVGGYGPGAGQQGPGSQGPGSGGQQGPYGPSSSTAAASAGGYGPGTVQQGPRSQAPVASAA
ASRLSSPQASSRVSSAVSTLVSSGPTNPAALSNAISSVVSQVSASNPGLSGCDVLVQALLEIVSALVHILGSSSIG
QINYAASSQYTQMVGNSVAQALG

>Aur_MaSp3a
MAWIARLPLLVLVALCTQTMIVHGQDSHPWKDTRTTESFMENFVEYFRQSGYFNSDDIESIKDLADTLIQSL
NEMQAKGKNSHQVLQALNMGFAAGVAELVNSDGINLKEKQNAIREAMKKSQLQTTGVINESFMNEMDKL
MQMFSQINALNDDSVGY**GAGA**VSYASSASASNAQGIGQNFGYQGQGSSSSSVSSISVGGLPQGPVGSDSY
EYSLSVNSLSGLPNAYGGQYDSGVGMEQGSLGTGSSGGAAVATASGGASGNGYGPGYGGIGGFGLGGTSA

AVAVGRAGESGYGRGGNRLGGVRAAASSGVGPGGPGYGGDGYSGTGRSGPGVAAAAAASGGRGGGDRY
GPLGAGGYGQGSGSGTGGAAAAAASSGEGPGGAGYSGDGYGGPGGSGPGSAAAAAASGGRGGDGRYG
QQGAGGYGQGGNGLGGAGAAATSGEGPGGAGYGGPGGSGPGSAAAAAASGGRGGGGRYGQQDADGY
GQGGSGLGGVGAAANAASSDEGPGGAGYGGEGGSGSGSAAAAAASGGQGGGGRYGPQGVGGYGQGG
SGLGGAGAAASSGEGPGGAGYGGDGYGGPGGSGPGSAAAAAVSGGRGGSGRYGQQGAGGYGQGNGSE
AAGAAAAAASSGGGPGGAGYGGQGGTGPGSAASAATSGGRGGGGRYGPQGAGGYGQGGNGLGGAGA
AASSGEGPGGAGYGGPGGSGPGSAAAAAVSGGRGGAGRYGQQGADGYGQGGSGLGGAGAAAAAASSG
EGPGDAGYGDDGYGGPGGSGPGSAAAAAASGGRGGDGRYGQQGAGGYGQGGNGLGGAGAAASSGEG
PGGAGYGGPGGSGPGSAAAAAASGGRGGGGRYGLQGAGGYGQGGSEIGGVGAAASAASSGEGPGGAGY
GGDGYGGPGGSGPGSASAAAASGGRGGFGGYGPQGARGYGQGGSGLGGVGAAASAASSVEGPAGAGYV
GDGYGGPGWSGPGTAAAAAASGGRGGGRRYGQQGAGGYGQGNGSEGAGAAAAAASSSEGPGGAGYG
GDGYVGQGGSGPGGAASAASALGGQGVGGGYGQQGAGGYGEGGSGSGGSGAAAAAASSGEGQGGAR
YSGDGYAVPGGSGQDGTASAAASASGIRGPGGLRGSkeiikkiivhrrvgsasdaeasvieenGYGGEGGYGAGYD
GQGGSAPGGAAAAAASGGQGGGSGYGPLGAGGYGQGGSGLGGAGAAAAAALSGEGPGGAGYGGPGLS
GPGSAAAAAASGGRGGGGRYGQQGAGGYGQRGSGLGGEGSAAAAASSGEGSGGAGYGGDGYGGPGGS
GLGAAAAAAASGGRGGGGRYGQQGAGGYGQGNGSEGAGASAAAASSGEGPGGAGYGGQGGSGPGSA
ASAAALGGRGGGGRYGPQGAGGYGQGGSGLGGAGEGAAAAAASSGEGPGGIDNGGDGYGGPGGSGPG
SAAAAAASGGQVGGSRYGPQGAGGYGQRSGSVRARAAAAAASSFEISGGAGYDGQVVSGPGSAAAAAAS
GGRGSGGRYGPQGADGYGQGSGSEGAGAAAAAASSSEGPGGAGYRGQGRSGSGSAAAAAASGGQGGG
GRYGPQGAGGYGQRGSGLGGAGAAAAAVSSGQGPGGADYGRDAYGGPEGSGPGSAAAAAASGGRGGG
GRYGPQGAGGYGPGGAGVAAAAASSGEGPGGAGYGGDGYGGPGGNGPDNAAAAAASGGRGGGGRYG
PQGAGGYGQGGSGLGEGAGEGGAGAAASSGEGPGGAGYDDDGYGGPGGSGPGSAAAAAASGGRGGG
GRYGQQGAGGYGQGGSGLGGVGAATSAASSGEGPGGGGYGGEGGSVPGSAAAAAASGGQGGGGRYGP
QGAGGYGEGGSGLGGAGAAAAAASSVEGPGGAGYDDDGYGAPGGSGPGSAAAAAASGGRGGGGRYGQ
QGAGGYGQGGSGLGGVGAATSAASSGEGPGGGGYGGEGGSGPGSAAAAAASGGREGGGRYGPQGAGG
YGQGGSGLGGAGAAASSVEFPGGAGYGDDGYGGPGGSEGNGVASAGTSSNGGPVELGSGRRGGSGLGG
ALSSAASTGGFSGPGGLRGPkeiikkiivhrrlgsasdasasvieenLYGPEAIGYYGQGGRGAGGAGAASAAVSSSE
GPGGVGYGGQLGSGSGSAAAAAPLGGRGGGGRYGAEGAGGFGKGGGGFGGAAAAASGLGPGENGFDA
AGYGGDGEAGPEGAAAAAAASGGGGSYGPQGAGGYGEGGSGSIGAGAAASAASSSGPGGASYGGQGES
GPGGAAGAAAAAAGGRGGRGRYGAYGAGGYGEGGSGSGGTGGFGSGSDVYGGQGGSGEGAAAAAAVE
SSGQGGRGRLGSNGALGARAAGAAASSGVGSGGAGYGGDGYDGQGGSEGNGAAAAAASSDEGPGGFRP
AVRGGSGQGGAVSSAASAGGRAGRDRLGSQGAGGAGGFDYGRDGYGGQGGSLEGGAAAAAAAGSAGY
GPQGAGDYGQGGSGPGGNGAAAASASSTAASVASRLSSPATLSRVSSAVSLFLDDDLDYPVAFSNAFDNVV
SGITLSYSNISGCELLVQSLMEVLSAVLGTAYGLNANSSVDIVRSVVNRFDY

>Aur_MaSp3b
MAWIARLPLLVLVALCTQTMIVHGQDSHPWKDTRTTELFFENFVECIRQSSYFNSEDIESIKVLAETLIQSLNG
MQAKGKTSHQMLQALNMGYAAGVAELVNSDGSNLQEKRNAIREAMKKSLLQATGVVNESFMNEMDKL
MQMFSQINGLNDDSGGYGAGAVSYASSASASNAQGIGQNFGYQGQGSSSSSVSSISVGGLPQGPVGSGTY
EYSLSVNSLSGSPSGYGGQYVRGVGVGQGGFASGAGGAAAATTSGGASGNGYGFGYGGIGGTGLGGASA
AVAVGIAGQGGYGQEGKGLERTGAAASSSVGPEGAIYGGEGYDGQGGSGPSGTAAAATASDGQGGGGRY
GPQGAGGYGEGGSGSGGAAAAAASSGVGPGGAGYGGDGYGGQGGSGPGGAAAAAGRGGDGRYGQQG
FGGFGQRGSETGGAGAAAAAASSGEGPGGTGYGGIEGSGPGGAASAAAASGGRGAGGRYGPEGSGGYG
QGGRGSGGAGAAASSGVGPGGAGYGGDGYGGQGGSGPGSAASAAAASDGEGGRGRYGQQGSGGYGQ

RGSGSGEASASAAAASSGGGPGGAGYGGDGYGGQGGSGPGGAAASAASGGRGGRYGQQGFGGFGQG
GSETLGAGAAAAAASSGEGSGGAGYGGIGRSGPGGAASAAAGGRGAGGRYGPEGSGGYGQGGRGSGLA
GAAASSGVGQGGAGYGGDDYGGQGGSGPVSAASAAAASDEQGGDGRYGQQGSGGYGQGGSGSGGAS
ASAAAASSGEGPGGAGYGGDGYGGQGGSGPGGAAASAASGGRGGRYGQQGFGGFGQGGSETGGAG
AAAAAASSGEGPGGAGYGGIGGSGPGGAASAAAASGGRGAGGRYGPEGSGGYGQGGRGSGGASAAASS
GVGPGGAGYGGDGYGGQGGSGPGSAASAAAASDGQGGRGRYGQQGSGGYGQGGSGSGGASASAAAA
SSGEGPGGAGYGGDGYGGQGGSGPVGAAASAASGGRGGRYGQQGFGGFGQGGSETGGAGAAAAAAS
SGEGPGGAGYGGIGGSGPGGAASAAAASGGRGAGGRYGPEGSGGYGQGGRGSGGAGAAASSGVGPGG
AGYGGDGYGGPGGSGPGSAASAAAASDGQGGRGRYGQQGSGGYGQGGSGSGGASASAAAASSGEGPG
GAGYGGDGYGGQGGSGPGGAAASAASGGRGGRYGQQGFGGFGQGGSETGGAGAAAAAASSGEGPG
GAGYGGIEGSGPGGAASAAAASGGRGAGGRYGPEGSGGYGQGGRGSGGAGAAASSGVGPGGAGYGGD
GYGGQGGSGPGSAASAAAASDGQGGRGRYGQQGSGGYGQGGSGSGGASASAAAASSGEGPGGAGYGG
DGYGGQGGSGPGGAAASAASGGRGGRYGQQGFGGFGQGGSETGGAGAAAAAASSGEGPGGAGYGGI
EGSGPGGAASAAAASSGRGARGRYGPEGSGGYGQGGRGSGGAGAAASSGVGPGGAGYGGDGYGGPGG
SGPGSAASAAAASDGQGGRGRYGQQGLGGYGQGGSGSGGASASAAASLSGEGPGGTGYGGDGYDGQV
GSGPAGTAASAASGGRGGRYGQQGFGGFGQGGSETGGAGAAAAAASSGEGPVGAGYGGIEGSGPGGA
ASAAAASGGRGARGRYGPEGSGGYGQGGRGSGGASAAASSGVGPGGAGYGGDGYGGQGGSGPGSAAS
AAAASDGQGGRGRYGQQGSGGYGQGGSGSGGASASAAAASSGEGPGGTGYGGDGYGGQGGSGPGGA
AASGGRGGRYGLQGFGGFGQGGSETGGAGAAAAAASSGEGPGGAGYGGIEGSGPGGAASAAAASSRR
GARGRYGPEGSGGYGQGGRGSGGASAAASSGVGPGGAGYGGDGYGGQGGSGPGSAASAAAASDGQGG
RGRYGQQGSGGYGQGGSGSGGASASAAAASSGEGPGGAGYGGDGYVGQGGSGPGGAAASAASGGRGG
GRYGQHGFGGFGQGGSETGGAGAAAAAASSGEGPGGAGYGGIGGSGPGGAASAAAASGGRGAGGRYG
PEGSGGYGQGGRGSGGASAAASSGVGPGGAGYGGDGYGGQGGSGPGSAASAAAASDGQGGRGRYGQQ
GSGGYGQLGSGSGGASASAAAATSGEGPGGTGYGGDGYGGQGGSGPGGEASAASASGGQGGGGYGQ
QGAGDYDQGGSGSGGSGAAAAAASSGEGPGGARYGGDGYAVQGGSGQDGIASAAASASGIGGPGGLRG
SkeiikkiivhrrvgsasdaeasvieenGYGGQGGYGAGYDGQGGSAPGGEAAAASGGRGGGGRYSSQGAGRYG
EGGSGSRGAGAAASAASSSGTGGTGYSGQGVSGPGGAASAAAAASGGRGGRGRYNAEGAGGYGEGRNE
SGGTGGFGSGSDGYGEQGESGGSGAAAAAGSAGHGPQGAGDYGQGGSGSGGNEAAAASSTAASVASRL
SSPAALSRVSSAVSVFLDDDLDYPVAFTNAFDNVVSGITLSNSDISGCELLVQSLMEVLSAVLGTAYGLNANSS
VDIVRSVVNRFDY

>L.hesperus_MaSp1
MTWSTRLALSFLFVLCTQSLYALAQANTPWSSKANADAFINSFISAASNTGSFSQDQMEDMSLIGNTLMAA
MDNMGGRITPSKLQALDMAFASSVAEIAASEGGDLGVTTNAIADALTSAFYQTTGVVNSRFISEIRSLIGMFA
QASANDVYASAGSSGGGGYGASSASAASASAAAPSGVAYQAPAQAISFTLRGQQPVSYGQGGAGAGAA
AAAAAAAGGAGQGGQGGYGQGGYGQGGAGQGGSGAAAAAAAAAGGTGQGGAGQGGAGAAAAAAA
AAGGAGQGGQGGYGQGGYGQGGTGQGGAGAAAAAAAGGAGQGGQGGYGQGGYGQGGYGQGGS
GAAAAAAAAAGGAGQGGQGGYGQGGYGQGGAGQGGAGAAAAAAAAAGGAGQGGYGRGGAGQGGA
AAAAAAAAGAGQGGYGGQGAGQGGSGAAAAAAAAGGAGQGGQGGYGQGGYGQGGSGAAAAAAAA
GGAGQGGQGGYGQGGYGQGGAGQGGAGAAAAAAAGGAGQGGQGGYGQGGYGQGGAGQGGAGA
AAAAAAAGGAGQGGQGGYGQGGYGQGGAGQGGAGAAAAAAAGGAGQGGQGGYGQGGYGQGGG
QGGAAAAAAAAAGGAGQGGYGRGGAGQGGAAAAAGAGQGGYGGQGAGQGGAGAAAAAAAGGAG
QGGQGGYGRGGYGQGGAGQGGAGAAAAAAAGGAGQGGQGGYGQGGYGQGGAGQGGAAAAAAA
AGGAGQGGYGRGGAGQGGAAAAAAAAAGAGQGGYGGQGAGQGGAGAAAAAAAAGGAGQGGQGDY

GRGGYGQGGAGQG**GAGA**AAAAAAAGGAGQGGQGGYGQGGYGQGGAGQGGAAAAASAAAAGGAGQ
GGYGRGGAGQGGAAAAAGAGQGGYGGQGAGQG**GAGA**AAAAAAAGGAGQGGQGGYGRGGYGQGGA
GQG**GAGA**AAAATAAGGAGQGGQGGYGQGGYGQGGAGQGGAAAAAAAAAGGAGQGGYGRGGAGQ
GGAAAAAAAAAGAGQGGYGGQGAGQG**GAGA**AAAAAGGAGQGGQGGYGRGGYGQGGAGQG**GAGA**
AAAAAAAGGAGQGGQGGYGQGGYGQGGAGQGGAAAAAAAAAGGAGQGGYGRGGAGQGGAAAAAG
AGQGGYGGQGAGQG**GAGA**AAAASRGAGQGGQGGYGRGGYGQGGAGQG**GAGA**AAAAAAAGGAGQ
GGQGGYGQGGYGQGGAGQGGAAAAAAAAGGAGQGGYGRGGAGQGGAAAAAGAGQGGYGGQGAG
QG**GAGA**AAAAAAAGGAGQGGQGGYGRGGYGQGGAGQG**GAGA**AAAAAAAGGAGQGGQGGYGQGG
YGQGGAGQGGAAAAAAAAAGGAGQGGYGRGGAGQGGAAAAAAAAAGSGQGGYGGQGAGQG**GAGA**
AAAAAAAGGAGQGGQGGYGRGGYGQGGAGQG**GAGA**AAAAAAAGGAGQGGQGGYGQGGYGQGGA
GQGGAAAAAAAAAAGGAGQGGYGRGGAGQGGAAAAAGAGQGGYGGQGAGQG**GAGA**AAAAAAAGG
AGQGGQGGYGRGGYGQGGAGQGGAGTAAAAAAAGGAGQGGQGGYGQGGYGQGGAGQGGAAAAA
AAAAGGAGQGGYGRGGAGQGGAAAAAAAAAGAGQGGYGGQGAGQG**GAGA**AAAAAAAGGAGQGGQ
GGYGRGGYGQGGAGQG**GAGA**AAAAAAAGGASQGGQGGYGQGDYGQGGAGQGGAAAAAAAAGGAG
QGGYGRGGAGQGGAAAAAGAGQGGYGGQGAGQG**GAGA**AAAAAAAGGAGRGGQGGYGRGGYGQGG
AGQG**GAGA**AAAAAAAGGAGQGGQGGYGQGGYGQGGTGQGGAAAAAAAAAGGAGQGGYGRGGAGQ
GGAAAAAAAAAGAGQGGYGGQGAGQG**GAGA**AAAAAAAGGAGQGGQGGYGRGGYGQGGAGQG**GA**
GAAAAAAAAGGAGQGGQGGYGQGGYGQGGYGQGGAGQGGAAAAAAAAGGAGQGGYGRGGAGQG
GAAAAAGAGQGGYGGQGAGQG**GAGA**AAAAAAAGGAGQGGQGGYGRGGYGQGGAGQG**GAGA**AAA
AAAAGGAGQGGQGGYGQGGYGQGGAGQGGAAAAAAAAAGGAGQGGGRGGAGQGGAAAAAAAAAG
SGQGGYGGQGAGQG**GAGA**AAAAAAAGGAGQGGQGGYGRGGYGQGGAGQG**GAGA**AAAAAAAGGAG
QGGQGGYGQGGYGQGGYGQGGAGQGGAAAAAAAAAAGGAGQGGYGRGGAGQGGAAAAAGAGQG
GYGGQGAGQG**GAGA**AAAAAAAGGAGQGGQGGYGRGGYGQGGAGQG**GAGA**AAAAAAAGGAGQGG
QGGYGQGGNGQGGAGQGGAAAAAAAAGGAGQGGYGRGGAGQGGAAAAAAAAAGAGQGGYGGQG
AGQG**GAGA**AAAAAAAGGAGQGGQGGYGRGGYGQGGAGQG**GAGA**AAAAAAAGGASQGGQGGYGQ
GDYGQGGAGQGGAAAAAAAAGGAGQGGYGRGGAGQGGAAAAAGAGQGGYGGQGAGQG**GAGA**AA
AAAAAGGAGRGGQGGYGRGGYGQGGAGQG**GAGA**AAAAAAAGGAGQGGQGGYGQGGYGQGGAGQ
GGAAAAAAAAAGGAGQGGYGRGGAGQGGAAAAAGAGQGGYGGQGAGQG**GAGA**AAAAAAAGGAGR
GGQGGYGRGGYGQGGAGQG**GAGA**AAAAAAAGGAGQGGQGGYGQGGYGQGGAGQGGAAAAAAAA
VGGAGQGGYGRGGAGQGGAAAAAAAAAAGSGQGGYGGQGAGQG**GAGA**AAAAAAAGGAGQGGQGG
YGGGYGQGGAGQG**GAGA**AAAAAAAGGAGQGGQGGYGQGGYGQGGAGQGGAAAAAAAAAGGAG
QGGYGRGGAGQGGAAAATGAGQGGYGGQGAGQG**GAGA**AAAAAAAGGAGQGGQGGYGRGGYGQGG
AGQG**GAGA**AAAAAAGGAGQGGQGGYGQGGYGQGGAGQGGAAAAAAAAGGAGQGGYGRGGAGQG
GAAAAAAAAAGAGQGGYGGQGAGQG**GAGA**AAAAAAAGGAGQGGQGGYGRGGYGQGGAGQG**GAG**
AAAAGGAGQGGQGGYGQGGYGQGGAGQGGAAAAAAAAAAGGAGQGGYGGYG**QQ**G**GAGA**AAAAAS
GPGQIYYGPQSVAAPAAAAASALAAPATSARISSHASALLSNGPTNPASISNVISNAVSQISSSNPGASACDVL
VQALLELVTALLTIIGSSNIGSVNYDSSGQYAQVVTQSVQNAFA

>L.hesperus_MaSp2
MTTMNWSTRLVLSILVVLCTQSLCALGQANTPWSSKENADAFIGAFMNAASQSGAFSSDQIDDMSVISNTL
MAAMDNMGGRITQSKLQALDMAFASSVAEIAVADGQNVGAATNAISDALRSAFYQTTGVVNNQFITGISS
LIGMFAQVSGNEVSYSSAGSSSAAASEAVSAGQGPAAQPVYAPSGASAAAAAASGAAPAI**QQ**AYERGGSGS
AAAAAGSGPSGYGQGAGGPG**GAGA**AAGAAAAGGSGPGGYGQGPAAYGPSGPSG**QQ**GYGPGGSGAAA
AAAAAAGSGPSGYGPGAGGPG**GAGA**AAAAAAAGGSGPGGYGQGQASYGPSGPSG**QQ**GYGPGGSGAAA

AAAAAAGSGPSGYGPGAAAAAAAGSAGPGTQQGYGPGGSGAAAAAGSGPRGYGPRGPGGAGAAATAA
RGSGPGGYGQGPAGYGTSGPSRQQGYGPGGSGAAAAAAAAAGGAGPGRQQGYGPGGSGAAAATAAGG
PGYVGQQRYGPGGAGAAAAAAAGSAGPSRQQAYGPGGSGPAAATAAAGSGPSGYGPGASGPVGADAA
AAAATGSAGPGRQQAYGPGESGAAAAAASGAGPGRQLGYGPGGSGAAAAAAAGGPGYGGQQGYGPGG
AGAAAAAAAGGAGPGRQQTYGPGGSGAAATAAGGSGPGGYGQGPSGYGPSGPGGQQGYGPGGSGAA
AAAAAGEAGPGRQQGYGPRGSGAAAAAAAGGPGYGGQSGYGPGGAGAAAAAAAGGAGPGRQQEYGP
GGSGAAAAAAAAAGSGPSGYGPGAAGPIGPGGAGAAAGGSGPVGYGQGPSGYGASGTGGEQDYGPG
GSGAAAAAAAAAGGAGPGRQQGYGPGGSGAAAAAAAGGPGYGGQQGYGPGGAGAAAAAAAGGAGP
GRQQPYGPGGAGAAAAAGGSGPGGYGQGPSGYGASGPGGQQGFGPGGSGAAAAAAAGGAGPGRQQ
GYGPGGSGAAAAAGGTGYGGQQGYGPGGAGAAAAAAAGAGPGRQQEYGPGGTGAAAAAAAAAGSG
PSGYGQGAAGPSGPGGEGTAAAAAAAGGSGPGGYGQGPSGYSASGPGGQQGYGPGGSGLAAAAAAAA
GGAGTGRQQGYGPGGSGAAAAAAAVGPGYGGQQGYGPGGAGAAAAAAAGGAGPGRQQAYGPGGSG
ATAAAAVAGSGPSGYGPGGAGAAAAAAAGGAGPGRQQAYGPGGSGAAAAAASGAGPGRQQVYGPGGS
GAAAAAAAGGPGYGGQQGYGPGGAGAAAAAAAGGAGQGTRQAYGPGGSGAAAAAAGPGPSGYGPGA
AGPSGPGLAGAAAAAAAGGSGPGGNGQRPSGYGQSGTGGQQGYGPGGSGAAAAAAAAAGGAGPGRQ
QGYGPGSSGAAAAAAAGGPGYGGQQGYGPGGAGAAAAAAAGGAGPGTQQAYGPGGSGAAAAAAAAA
GGAGPGRQQGYGPGSSGAAAAAAAGGPGYGGQQGYGPGGAGAAAAAAAGGAGAGRQQAYGPGGSG
AAAAGSGPSGYEPGAAGPGGAGAAAAAAVGAGPGRQQAYGQGGSGAVAAAAAGGPGYGGQQGYEQ
GGAGAASAAAAGGEGPARQQAYGPGGSGAAAAAAGGAGPGRQQGYGPGSSGAAAAAAAGGPGYGGQ
QGYGPGGAGAAAAAAAGGAGPGRQQAYGPGGSGAAAAAAAGTGPSGYGPGAAGPGGAGAAAAAAAG
SAGPGRQQAYGPGGSGAAAAAAAGGPGYGGQQGYGPGGAGAAAAAAAGGAGPGTQQAYGPGGSGAA
AAAAAAAGGAGPDRQQGYGPGSSGAAAAAAAGGPGYGGQQGYGPGGAGAAAAAAAAGPGPSGYGP
GGAGAAAAAAAAGGSGPGGYGQGPSGYSASGPGGQQGYGPGGSGLAAAAAAAAGGAGTGRQQGYGP
GGSGAAAAAAAVGPGYGGQQGYGPGGAGAAAAAAAGGAGPGRQQAYGPGGSGATAAAAAAGSGPSG
YGPGGAGAAAAAAAGGAGPGRQQAYGPGGSGAAAAAASGAGPGRQQVYGPVGSGAAAAAAAGGPGY
GGQQGYGPGGAGAAAAAAAGGAGQGTRQAYGPGGSGAAAAAAGPGPSGYGPGAAGPSGPGLAGAAA
AAAAGGSGPGGNGQRPSGYGQSGPGGQQGYGPGGSGAAAAAAAAAGGAGPGRQQGYGPGSSGAAAA
AAAGGPGYGGQQGYGPGGAGAAAAAAAGGAGPGTQQAYGPGGSGAAAAAAAAAGGAGPGRQQGYG
PGSSGAAAAAAAGGPGYGGQQGYGPGGAGAAAAAAAGGAGAGRQQAYGPGGSGAAAAGSGPSGYES
GAAGPGGAGAAAAAAVGAGPGRQQAYGQGGSGAVAAAAAGGPGYGGQQGYEQGGAGAASAAAAG
GEGPARQQAYGPGGSGAAAAAAGGAGPGRQQGYGPGSSGAAAAAAAGGPGYGGQQGYGPGGAGAAA
AAAAGGAGPGRQQAYGPGGSGAAAAAAAGTGPSGYGPGAAGPGGAGAAAAAAAGGAGPGRQQAYGP
GGSGAAAAAAAGGPGYGGQQGYGPGGAGAAAAAAAGGAGPGTQQAYGPGGSGAAAAAAAAAGGAG
PDRQQGYGPGSSGAAAAAAAGGPGYGGQQGYGPGGAGAAAAAAAAGPGPSGYGPGGAGAAAAAAA
AGGSGPGGYGQGPSGYGPSGPGGQQGNGPGGSGAAAAAAAAAGGAGPGRQQGYGPGGAAAAAAAGG
PGYGGQQGYGPGGAGAAAAAAAGGAGPGRQQAYGPGGAGAAAAAAAAGPGPSGYGPGASGPSGTG
GAGAAAAAAAAGGSGPGGYGQGASGYGPSGPGGQQGYGPGGSGAAAAAAAAAGGAGPGRQQGYGPG
SSGAAAAAAAGGPGYGGPQGYGPGGAGAAAAAAAGGAGPGRQQAYGPGGSGAAAAAAGSGPSGYGPG
AAGPGGTGAAAVAAAGGAGPGRQQAYGPGGSGAAAAAAAGGPGYGGQQGYGPGGAGAAAAAAGG
AGPGTQQLYGPGGSGAAAAAAAAAGSGPSGYGPGAAGPSGPGGAGAAAAASAGGSGPGGYGQGPSG
YGPTGPVGQQGYGPGGSGAAAAAAAAAGGAGPGRQQGYGPGSSGAAAAAAAGGPGYGGQQGYGPGG
AGAAAAVAAGGAGPGRQQGYGPGSSGAAAAAAAGGPGYGGQQGYGPGGAGAAAVAAGGAGPGRQ
QGYGPGSSGAAAAAAAGGPGYGGQQGYGLVAGAAAAVAAGGAGPGRQQAYGPGGSGAAAAAAAGS
GRSGYGPGAAGTGGAGAAAAAAAGGAGSGRQQAYGPGGSGAAAASAAGGPGYGGQQGYGPGGAGAA

AAAAAGGAGPGT**QQ**AYGPGGSGAAAAAAAASGPGPSGYEPGAAGPSGPA**GAGA**AAAAAAAGGSGPGG
YGQGPSGYGPSGPGG**QQ**GYGPGGSGAAAAAAAAAGGAGPGR**QQ**GYGQGSSGAAAAAAAGGPGYGG**Q**
QVYGPG**GAGA**AAAVAAGGAGPGR**QQ**AYGPGGSGAAAGSGPSGYGPGAAAAAAAGGAGPGR**QQ**AYGP
GGSGAAAAAAAGGPGYGG**QQ**GYGPG**GAGA**AAAAAAGGSGPGGYGQGPSGYGPSGSGGQGYGQGGSG
AAAAAAGGAGPGR**QQ**GYGPGSSGAAAAAAAGGPGFGG**QQ**GYGPGGSGAAAAAAAGGAGPGR**QQ**AYG
PGGSGAAAAAAAAAGSGPSGYGPSAAGPSGPGGSGAAGGSGPGGFGQGPAGYGPSGPGG**QQ**GYGPGAS
GAAAAAAASGSGGYGP**SQ**YVPSSVASSAASAASALSSPTTHARISSHASTLLSSGPTNAAALSNVISNAV**SQ**V
SASNPGSSSCDVLVQALLEIITALISILDSSSVGQVNYGSSGQYAQIVGQSM**QQ**AMG
